# JAGGED1/NOTCH3 activation promotes aortic hypermuscularization and stenosis in elastin deficiency

**DOI:** 10.1172/JCI142338

**Published:** 2022-03-01

**Authors:** Jui M. Dave, Raja Chakraborty, Aglaia Ntokou, Junichi Saito, Fatima Z. Saddouk, Zhonghui Feng, Ashish Misra, George Tellides, Robert K. Riemer, Zsolt Urban, Caroline Kinnear, James Ellis, Seema Mital, Robert Mecham, Kathleen A. Martin, Daniel M. Greif

**Affiliations:** 1 Yale Cardiovascular Research Center, Section of Cardiovascular Medicine, Department of Internal Medicine,; 2 Department of Genetics,; 3 Department of Pharmacology, and; 4 Department of Surgery, Yale University, New Haven, Connecticut, USA.; 5 Congenital Division, Department of Cardiothoracic Surgery, Stanford University School of Medicine, Stanford, California, USA.; 6 Department of Human Genetics, Graduate School of Public Health, University of Pittsburgh, Pittsburgh, Pennsylvania, USA.; 7 Genetics and Genome Biology,; 8 Developmental and Stem Cell Biology, The Hospital for Sick Children, Toronto, Ontario, Canada.; 9 Department of Cell Biology and Physiology, Washington University School of Medicine, St. Louis, Missouri, USA.

**Keywords:** Vascular Biology, Cardiovascular disease

## Abstract

Obstructive arterial diseases, including supravalvular aortic stenosis (SVAS), atherosclerosis, and restenosis, share 2 important features: an abnormal or disrupted elastic lamellae structure and excessive smooth muscle cells (SMCs). However, the relationship between these pathological features is poorly delineated. SVAS is caused by heterozygous loss-of-function, hypomorphic, or deletion mutations in the elastin gene (*ELN*), and SVAS patients and elastin-mutant mice display increased arterial wall cellularity and luminal obstructions. Pharmacological treatments for SVAS are lacking, as the underlying pathobiology is inadequately defined. Herein, using human aortic vascular cells, mouse models, and aortic samples and SMCs derived from induced pluripotent stem cells of *ELN*-deficient patients, we demonstrated that elastin insufficiency induced epigenetic changes, upregulating the NOTCH pathway in SMCs. Specifically, reduced elastin increased levels of γ-secretase, activated NOTCH3 intracellular domain, and downstream genes. *Notch3* deletion or pharmacological inhibition of γ-secretase attenuated aortic hypermuscularization and stenosis in *Eln^–/–^* mutants. *Eln^–/–^* mice expressed higher levels of NOTCH ligand JAGGED1 (JAG1) in aortic SMCs and endothelial cells (ECs). Finally, *Jag1* deletion in SMCs, but not ECs, mitigated the hypermuscular and stenotic phenotype in the aorta of *Eln^–/–^* mice. Our findings reveal that NOTCH3 pathway upregulation induced pathological aortic SMC accumulation during elastin insufficiency and provide potential therapeutic targets for SVAS.

## Graphical abstract

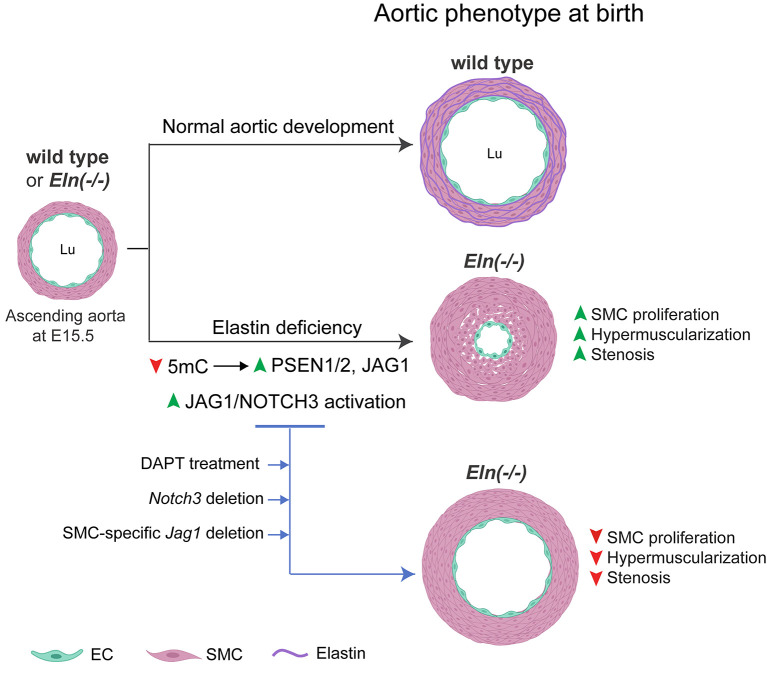

## Introduction

The vasculature is an intricately arranged network of blood vessels with vascular walls that deliver nutrients to, and remove waste products from, target organs. The arterial wall consists of an inner endothelial cell (EC) lining (tunica intima), smooth muscle cells (SMCs) supported by elastic lamellae (tunica media), and the outermost adventitial layer containing fibroblasts and connective tissue (tunica externa). Elastin is the major component of circumferential elastic lamellae that alternate with rings of SMCs to form lamellar units in the media of large elastic vessels such as the aorta. Excessive and aberrant accumulation of SMCs and cells derived from SMCs are a hallmark of diverse obstructive vascular diseases such as supravalvular aortic stenosis (SVAS), atherosclerosis, restenosis, vein graft failure, and pulmonary hypertension (1–6). These diseases, as well as physiological closure of the ductus arteriosus, are associated with enhanced SMC proliferation and abnormal or disrupted elastic lamellae structure (7–16). Indeed, SVAS, a devastating pediatric condition with obstruction of large- and medium-sized arteries, results from loss-of-function, hypomorphic, or deletion mutations of one elastin (*ELN*) allele (15, 17–19). Similar to SVAS patients, late-stage embryonic or early neonatal *Eln^–/–^* mice have increased vascular wall cellularity and arterial lumen obstructions (20). *Eln^+/–^* mice display thinner and higher numbers of elastic lamellae with additional SMC layers; however, in contrast with *Eln^–/–^* mice and SVAS patients, *Eln^+/–^* mice do not develop aortic stenosis (15, 21). SVAS occurs as an isolated entity (i.e., nonsyndromic) or as an integral part of Williams-Beuren syndrome (WBS), a multiorgan system disorder caused by heterozygous deletion of approximately 27 genes (including *ELN*) on chromosome 7 (19). Unfortunately, the mechanistic link between elastin defects and hypermuscularization in vascular diseases remains incompletely understood. As a consequence, there is a lack of pharmacological agents that prevent excessive proliferation and accumulation of SMCs, and major surgery remains the only therapy for vessel obstruction in elastin arteriopathy.

The evolutionarily conserved NOTCH signaling pathway plays key roles in diverse vascular developmental programs, such as aortic wall morphogenesis, EC tip-stalk dynamics during angiogenesis, and in select vascular diseases (22–30); however, to the best of our knowledge no prior investigations have evaluated the role of the NOTCH pathway in aortic hypermuscularization or stenosis in the context of elastin insufficiency. NOTCH signaling is initiated by the binding of transmembrane ligands and receptors on neighboring cells. In mammals, the repertoire of NOTCH ligands consists of Jagged (JAG1 and -2), Delta-like ligand 1, 3, and 4, and the receptors are NOTCH1–4. Signaling via JAG1 on ECs and SMCs is implicated in differentiation of arterial wall SMC layers (25–28), and NOTCH3 is highly expressed in human arterial SMCs (22). In *Notch3*-null mice, major elastic arteries of the trunk (e.g., aorta) are indistinguishable from those of wild-type (WT) mice, whereas smaller caliber arteries in mutants have thinner tunica media, impaired SMC differentiation, and incomplete vessel maturation (31). In regard to human diseases, cerebral autosomal dominant arteriopathy with subcortical infarcts and leukoencephalopathy (CADASIL) is caused by *NOTCH3* mutations (30), and enhanced NOTCH3 expression in SMCs of small arteries in the lung is associated with pulmonary hypertension (29).

In the current study, we report that NOTCH3 activation and signaling are upregulated in cultured human aortic SMCs (haSMCs) with *ELN* silencing, in the aortic media of both *Eln^–/–^* mice and WBS patients, as well as in WBS and nonsyndromic SVAS induced pluripotent stem cell–derived (iPSC-derived) SMCs. Upon engaging ligand, the transmembrane NOTCH receptor is cleaved by the enzyme γ-secretase, releasing the NOTCH intracellular domain (NICD) and liberating it to enter the nucleus and modulate gene expression (32). Our results indicate that elastin depletion results in increased SMC levels of JAG1, the γ-secretase complex, the activated form of NICD3, and downstream target genes. Mechanistically, SMCs lacking elastin have reduced global DNA methylation — an epigenetic mark that drives gene silencing (33, 34) — and decreased DNA methyltransferase 1 (DNMT1). In particular, elastin depletion in SMCs decreases DNA methylation mark at the promoters of key NOTCH pathway genes *JAG1* and γ-secretase catalytic subunits PSEN1 and PSEN2, facilitating their upregulation. Moreover, pharmacological inhibition of γ-secretase or genetic deletion of *Notch3* attenuates aortic hypermuscularization and stenosis in *Eln^–/–^* mice as well as abrogates excessive muscularization in *Eln^+/–^* mice. We previously reported that integrin β3 plays a key role in elastin aortopathy (3). Herein, our data indicate that NOTCH3 regulates integrin β3 expression in haSMCs and in mice, and NICD3 binds the promoter of *ITGB3*, the gene encoding integrin β3, and these effects are enhanced by elastin deficiency. The initial paper describing that *Eln* deletion results in arterial stenosis reported a lack of evidence for EC activation or damage (20). Interestingly, our results suggest that EC JAG1 protein is increased in *Eln^–/–^* mice at least partly through the effects of the extracellular matrix (ECM) deposited by elastin-deficient SMCs. Finally, *Jag1* deletion in SMCs, but not in ECs, mitigates the hypermuscularized and stenotic phenotype of the *Eln^–/–^* aorta. Taken together, our data in cultured human cells, mouse models, and samples from humans with SVAS and WBS are the first to our knowledge to implicate a role of the NOTCH signaling pathway and epigenetic remodeling in the pathogenesis of elastin aortopathy and to identify select NOTCH3 pathway members as attractive therapeutic targets for human SVAS and WBS. In addition, these studies provide fundamental mechanistic insights that are integral for advancing potential therapies for a cohort of proliferative and obstructive arterial diseases associated with impaired elastin.

## Results

### Upregulation of NOTCH3 pathway with loss of elastin.

**Figure 1:**
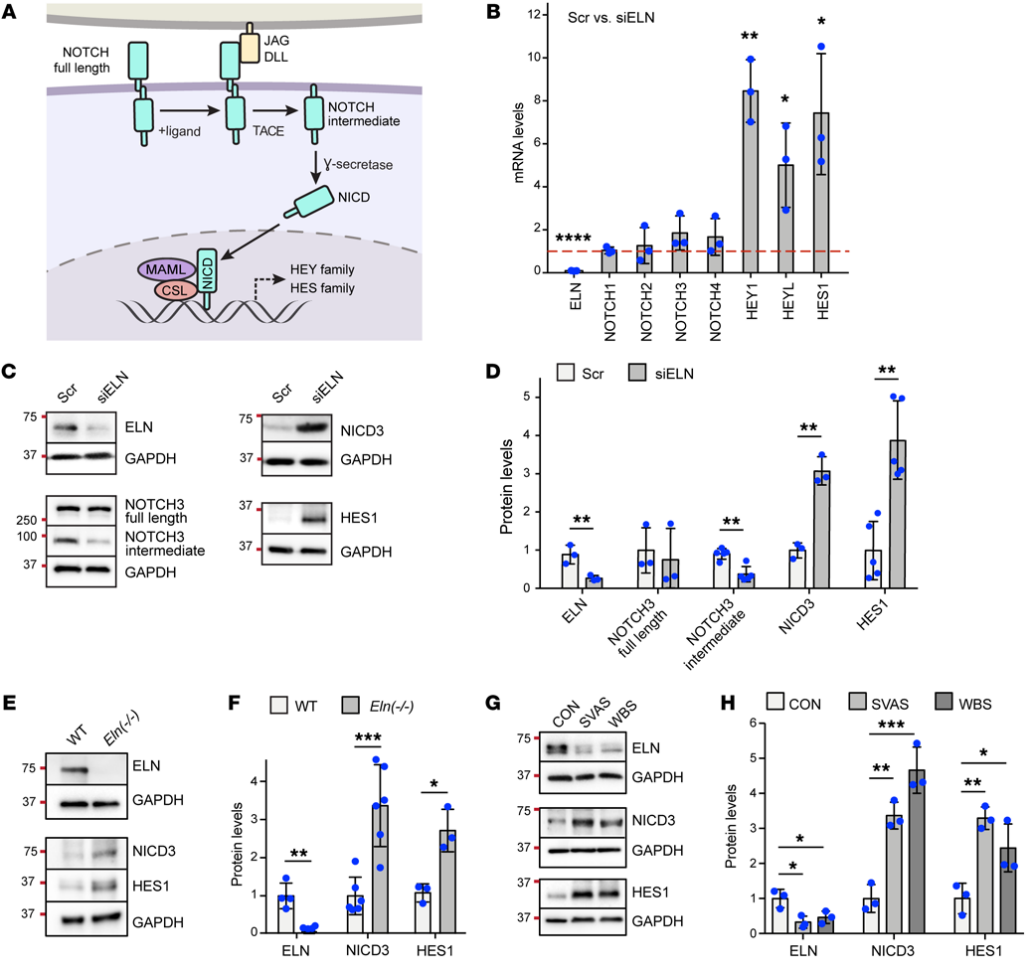
Upregulation of NOTCH3 pathway in human and mouse elastin mutants. (**A**) Schematic of the NOTCH3 pathway. Upon binding ligand (JAG or Delta-like ligand [DLL]) expressed by a neighboring cell, the transmembrane NOTCH full-length receptor is cleaved by tumor necrosis factor-α–converting enzyme (TACE), producing an intermediate form that remains membrane bound but lacks the extracellular region. This intermediate form is further cleaved by γ-secretase to release the NOTCH intracellular domain (NICD), which translocates to the nucleus, forms a complex with transcription factor CSL and coactivator Mastermind-like (MAML), and induces expression of target genes (e.g., HEY and HES family members). (**B**–**D**) haSMCs were treated with scrambled (Scr) or *ELN*-specific siRNA (siELN), and lysates were analyzed. In **B**, histogram depicts levels of indicated transcripts relative to *18S* rRNA in lysates as assessed by qRT-PCR and normalized to Scr treatment (*n =* 3). Western blots probed for ELN, NOTCH3 (full length and intermediate forms), NICD3, HES1, and GAPDH are shown in **C**, with densitometry of protein bands relative to GAPDH and normalized to Scr in **D** (*n =* 3–5). *P < 0.05, **P < 0.01, ****P < 0.0001 vs. Scr by Student’s *t* test. (**E** and **F**) Aortic lysates from WT or *Eln^–/–^* mice at P0.5 (2 aortas pooled per genotype for each *n*) were resolved by Western blotting for ELN, NICD3, HES1, and GAPDH (**E**), with densitometry of protein bands relative to GAPDH and normalized to WT (**F**). *n =* 3 to 6 mice. *P < 0.05, **P < 0.01, ***P < 0.001 vs. WT by Student’s *t* test. (**G** and **H**) iPSC-derived SMC progenitors from WBS or nonsyndromic SVAS patients or controls were differentiated into SMCs. Protein levels of ELN, NICD3, HES1, and GAPDH in these iPSC-SMCs were assessed by Western blotting (**G**), with densitometric analysis of ELN, HES1, and NICD3 normalized to GAPDH (**H**) (*n =* 3). *P < 0.05, **P < 0.01, ***P < 0.001 by 1-way ANOVA with Tukey’s post hoc test. All data are averages ± SD. Gels and blots for HES1 and GAPDH were run contemporaneously (**C**, **E**, and **G**).

The NOTCH pathway plays essential roles in SMCs during development and disease of the cardiovascular system (22), but to the best of our knowledge prior studies have not evaluated the role of NOTCH in aortic hypermuscularization or stenosis in the context of elastin insufficiency. Upon engaging ligand, the transmembrane full-length NOTCH receptor is cleaved by tumor necrosis factor-α–converting enzyme to produce the NOTCH intermediate form (Figure 1A). Subsequent cleavage of the intermediate form by the γ-secretase complex releases NICD into the cytoplasm which translocates into the nucleus, forms a complex with the transcription factor CSL and coactivator Mastermind-like, and thereby modulates gene transcription (22). We initially treated haSMCs with nontargeting scrambled (Scr) RNA or *ELN*-specific silencing RNA (siRNA) to query the effect of reduced elastin levels on NOTCH pathway members. In haSMCs, *ELN* silencing did not alter transcript levels of the 4 mammalian NOTCH receptors but did result in an approximately 4- to 8-fold increase in levels of key NOTCH pathway downstream gene products, including hairy and enhancer of split (HES) and hairy/enhancer-of-split related with YRPW motif protein (HEY) family members (Figure 1B). Among the NOTCH receptors, NOTCH3 is highly expressed in arterial SMCs and not detected in ECs (35–37). To dissect the effect of reduced elastin on NOTCH3 proteolytic processing and activation, lysates collected from *ELN*-silenced haSMCs were analyzed by Western blotting (Figure 1C). Elastin knockdown did not change full-length NOTCH3 levels (in agreement with transcript levels in Figure 1B) but resulted in an approximately 2-fold reduction in the NOTCH3 intermediate form, with an approximately 3- to 4-fold increase in NICD3 and HES1 (Figure 1, C and D). These data suggest that loss of elastin activates the NOTCH3 pathway by inducing the proteolytic cleavage of the NOTCH3 intermediate form.

We next confirmed the upregulation of NICD3 and HES1 with reduced elastin gene dosage in mice and human patient samples. For mice, aortas were isolated from WT and *Eln^–/–^* pups on postnatal day 0.5 (P0.5), and aortic lysates were analyzed by Western blotting. *Eln^–/–^* aortas had approximately 3-fold higher protein levels of NICD3 and HES1 as compared with WT aortas (Figure 1, E and F). NOTCH3 intermediate form did not change in *Eln^–/–^* aortas, which might reflect differences in rates of protein synthesis, cleavage, and/or degradation in cultured haSMCs versus in vivo (Supplemental Figure 1; supplemental material available online with this article; https://doi.org/10.1172/JCI142338DS1). Prior studies have shown that NOTCH3 and NOTCH2 have opposing functions in regulating SMC proliferation (38). Interestingly, NOTCH2 protein levels (full length, intermediate form, and NICD2) were not altered in *Eln^–/–^* aortas, indicating the specificity of NOTCH3 activation during elastin deficiency (Supplemental Figure 2). Furthermore, to assess the NOTCH3 pathway in human elastinopathy, iPSC-SMC progenitors derived from skin fibroblasts of human control, SVAS, or WBS patients were differentiated into SMCs (Supplemental Figure 3). NICD3 and HES1 protein levels were increased in iPSC-SMCs of WBS and nonsyndromic SVAS patients compared with those of controls (Figure 1, G and H). Taken together, these data demonstrate that reduced elastin in SMCs results in upregulated NOTCH3 pathway signaling.

### Loss of elastin upregulates γ-secretase complex in SMCs by modulating DNA methylation.

**Figure 2:**
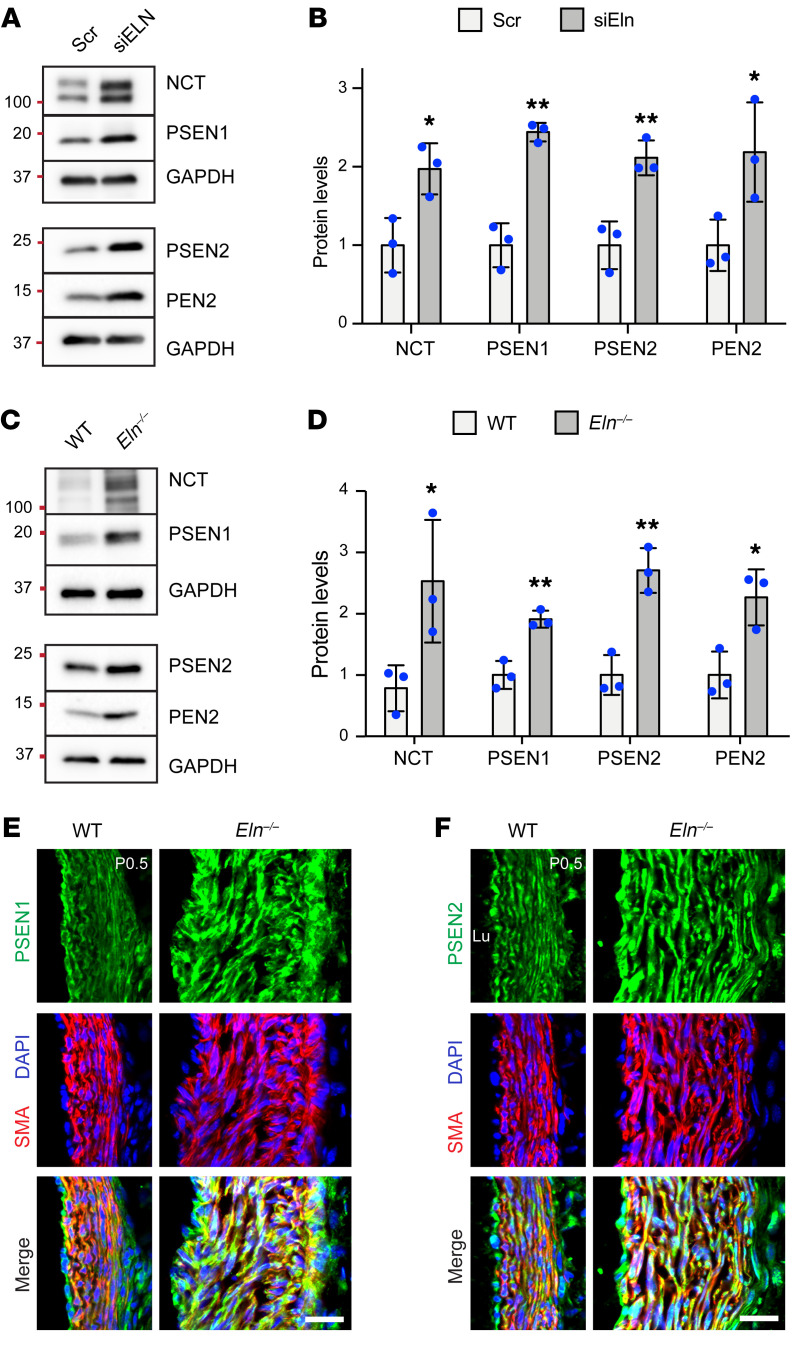
Enhanced aortic γ-secretase levels with elastin deficiency. (**A** and **C**) Lysates were analyzed by Western blotting for γ-secretase subunits NCT, PSEN1, PSEN2, and PEN2 and for GAPDH. In **A**, haSMCs were pretreated with Scr or siELN RNA, and in **C**, aortas of 2 WT or *Eln^–/–^* mice at P0.5 were pooled. (**B** and **D**) Densitometry of protein bands in **A** and **C** relative to GAPDH and normalized to Scr in **B** or WT in **D** (*n =* 3). *P < 0.05; **P < 0.01 by Student’s *t* test vs. Scr (**B**) or WT (**D**). Data are averages ± SD. (**E** and **F**) Ascending aortic cross sections of WT and *Eln^–/–^* mice at P0.5 stained for α-smooth muscle actin (SMA, marker of SMCs), nuclei (DAPI), and either PSEN1 in **E** or PSEN2 in **F**. *n =* 3 mice. Lu, lumen. Scale bars: 25 μm.

As suggested above, the changes in protein levels of the NOTCH3 intermediate form (reduced) and NICD3 (increased) in haSMCs with elastin silencing implicate γ-secretase–mediated proteolytic cleavage (Figure 1, A, C, and D). γ-Secretase is a proteasomal complex composed of multiple subunits, including the catalytic presenilins (PSEN1 or -2) and accessory subunits nicastrin (NCT) and presenilin enhancer 2 (PEN2) (39). We next assessed the levels of γ-secretase complex subunits in elastin-deficient haSMCs. siRNA-mediated elastin silencing upregulated protein levels of γ-secretase subunits NCT, PSEN1, PSEN2, and PEN2 (Figure 2, A and B). Similarly, aortic lysates from WT and *Eln^–/–^* pups at P0.5 had higher levels of the γ-secretase complex (Figure 2, C and D). Moreover, transverse cryosections of ascending aortas from WT and *Eln^–/–^* pups at P0.5 were stained for α-smooth muscle actin (SMA, marker of SMCs) and for PSEN1 or -2, confirming upregulation of the γ-secretase catalytic subunits (Figure 2, E and F). To assess the potential effect of sex on NOTCH3 pathway induction with elastin depletion, we compared the protein levels of NICD3, HES1, PSEN1, and PSEN2 in male and female *Eln^–/–^* pups. Sex-dependent differences in levels of these proteins or development of elastin aortopathy were not observed (Supplemental Figures 4 and 5). Overall, these data show that the NOTCH3 pathway is induced in *Eln^–/–^* aortas.

**Figure 3:**
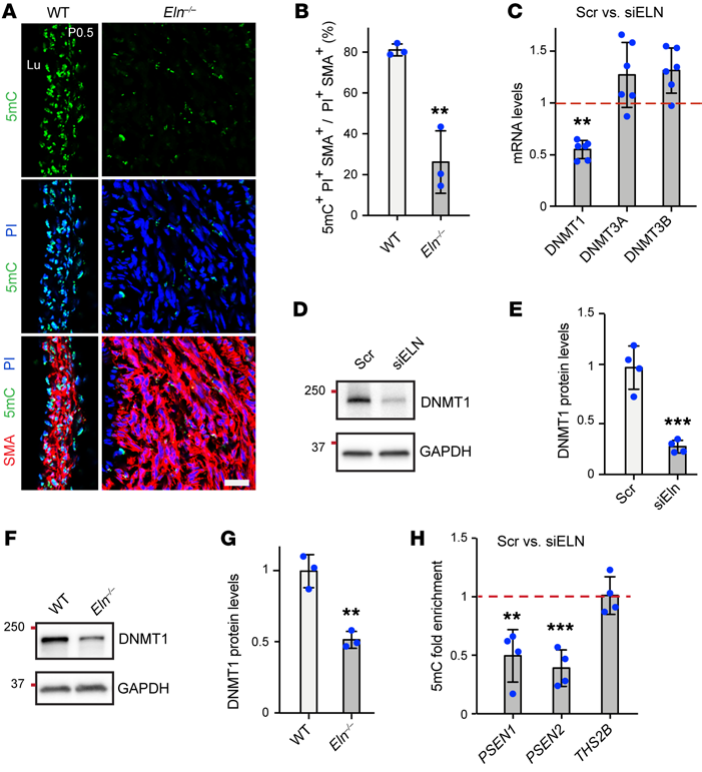
Elastin deficiency reduces DNA methylation (5mC) epigenetic mark and downregulates DNMT1 levels. (**A**) Transverse sections of ascending aorta from WT or *Eln^–/–^* pups at P0.5 stained for 5-methylcytosine (5mC), SMA, and propidium iodide (PI, nuclei). Lu, lumen. Scale bar: 50 μm. (**B**) Histogram representing the percentage of 5mC^+^ SMCs in **A** (*n =* 3 mice). **P < 0.01 vs. WT by Student’s *t* test. (**C**–**E**) haSMCs were pretreated with Scr or siELN and then lysates were analyzed. In **C**, histogram depicts transcript levels of *DNMT1*, *DNMT3A*, and *DNMT3B* from qRT-PCR relative to *18S* rRNA and normalized to Scr (*n =* 6). **P < 0.01 vs. Scr by Student’s *t* test. (**D**) Western blots for DNMT1 and GAPDH and densitometry of protein bands in **E** relative to GAPDH and normalized to Scr (*n =* 4). ***P < 0.001 vs. Scr by Student’s *t* test. (**F** and **G**) Aortic lysates from WT or *Eln^–/–^* mice at P0.5 analyzed by Western blotting for DNMT1 and GAPDH (**F**) with densitometry of protein bands relative to GAPDH and normalized to WT (**G**) (*n =* 3 mice). **P < 0.01 vs. WT by Student’s *t* test. (**H**) Genomic DNA was isolated from haSMCs pretreated with Scr or siELN and then subjected to methylated DNA chromatin immunoprecipitation (5mC ChIP). Histogram represents 5mC levels at the promoter regions of *PSEN1*, *PSEN2*, or *THS2B* (constitutive 5mC positive control) as assessed by qPCR and normalized to Scr (*n =* 4). **P < 0.01, ***P < 0.001 vs. Scr by Student’s *t* test. All data are averages ± SD.

Epigenetic modifications influence gene expression by altering chromatin accessibility and play a central role in regulating SMC behavior during vascular development and disease (33, 40). However, epigenetic changes that occur in SMCs in the context of elastin deficiency have not been reported to our knowledge. DNA methylation is a major form of chromatin remodeling in which DNMT-mediated transfer of a methyl group to the 5-C position of cytosine renders chromatin inaccessible and silences gene expression (41). Intriguingly, we found that excessive aortic SMCs in *Eln^–/–^* aortas display dramatically reduced 5-methylcytosine (5mC) mark, suggesting active chromatin remodeling and gene activation in elastin-depleted SMCs (Figure 3, A and B). Our analysis of major DNMTs (DNMT1, -3a, and -3b) in haSMCs revealed that elastin silencing results in a decrease in *DNMT1* transcript levels (Figure 3C). Similarly, Western blot analysis of lysates collected from *ELN*-silenced haSMCs or *Eln^–/–^* aortas demonstrated reduced DNMT1 protein levels (Figure 3, D–G). To investigate the role of modulated DNA methylation in expression of γ-secretase genes in SMCs with reduced elastin, we next assessed the status of 5mC at the promoter regions of the *PSEN1* and *PSEN2* genes. haSMCs were treated with Scr or *ELN*-specific siRNA (siELN) and then subjected to 5mC chromatin immunoprecipitation (5mc ChIP). Methylated DNA was immunoprecipitated with an anti-5mC monoclonal antibody, and recovered DNA was analyzed by quantitative real-time PCR (qPCR) to assess 5mC enrichment at the promoters of *PSEN1*, *PSEN2*, and *THS2B* (the latter being a positive control with constitutive 5mC mark). DNA methylation (5mC) was approximately 50% reduced at the *PSEN1* and *PSEN2* promoters in *ELN*-silenced haSMCs (Figure 3H), consistent with increased gene expression.

### Pharmacological γ-secretase inhibition attenuates aortopathy in elastin mutants.

**Figure 4:**
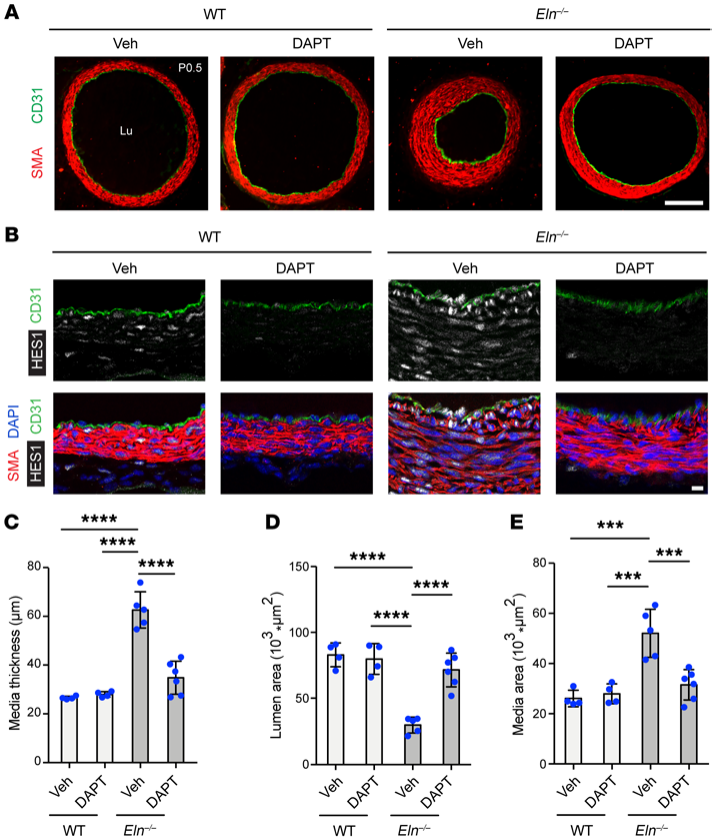
Inhibition of γ-secretase attenuates hypermuscularization and stenosis in *Eln^–/–^* mice. (**A**) Pregnant dams were injected on both E14.5 and E15.5 with either vehicle (4% DMSO in corn oil) or γ-secretase inhibitor DAPT (1 mg). Transverse sections of the ascending aorta from pups at P0.5 of indicated genotype and treatment were stained for SMA (SMC marker) and CD31 (EC marker). (**B**) Transverse sections in **A** stained for HES1, SMA, CD31, and nuclei (DAPI). Lu, lumen. Scale bars: 100 μm (**A**) and 10 μm (**B**). (**C**–**E**) Histograms represent medial thickness (**C**), lumen area (**D**), and medial wall area (**E**) of ascending aortas from **A**. *n =* 4 to 6 mice. ***P < 0.001; ****P < 0.0001 by 2-way ANOVA with Tukey’s post hoc test. All data are averages ± SD.

In the WT aorta, substantial levels of elastin (expressed as the soluble monomer tropoelastin) are initially detectable at embryonic day 14 (E14), and by approximately E15, the full complement of SMC layers, as in the adult mouse, are present (42, 43). *Eln*-null and WT aortas are indistinguishable at E15.5, but thereafter the *Eln^–/–^* aorta accumulates excess SMCs (3, 20). To test the hypothesis that γ-secretase and the NOTCH pathway, and specifically NOTCH3, are critical for aortic hypermuscularization and stenosis in *Eln^–/–^* mice, we next utilized pharmacological and genetic inhibition. The γ-secretase inhibitor DAPT (*N*-[(3,5-difluorophenyl)acetyl]-L-alanyl-2-phenyl]glycine-1,1-dimethylethyl ester) acts as a global NOTCH inhibitor by blocking cleavage of the membrane-bound NOTCH intermediate form and hence, the generation and release of the NICD is attenuated. Pregnant dams were injected with DAPT or vehicle on E14.5 and E15.5, and transverse sections of the ascending aorta in WT and *Eln^–/–^* pups at P0.5 were stained for CD31 (EC marker) and SMA (Figure 4A). Additionally, the aortas were stained with HES1 (downstream NOTCH target), confirming NOTCH pathway inhibition by DAPT treatment (Figure 4B). Consistent with prior studies (3, 20), *Eln^–/–^* newborns untreated or exposed to vehicle in utero displayed ascending aorta hypermuscularization and stenosis (Figure 4, C–E). However, DAPT treatment induced an approximately 2-fold reduction in medial thickness and wall area and an approximately 2-fold increase in lumen area of *Eln*-null mice without altering these parameters in WT pups. Furthermore, to assess the effect of DAPT-mediated NOTCH inhibition during elastin haploinsufficiency, we analyzed the *Eln^+/–^* aorta, which had an approximately 50% reduction in *Eln* transcript levels compared with WT (Supplemental Figure 6A) (15). In agreement with previous studies (15, 21), newborn *Eln^+/–^* aortas had a thicker media due to accumulation of additional lamellar units and SMC layers (Supplemental Figure 6B) without lumen occlusion and stenosis. Newborn WT or *Eln^+/–^* mice were injected daily with DAPT or vehicle from P2.5–P5.5, and aortas were analyzed on P7.5. DAPT treatment reversed the increased medial thickness and wall area seen in the early postnatal *Eln^+/–^* aortas (Supplemental Figure 7).

### Notch3 deletion attenuates elastin aortopathy and excessive SMC proliferation in elastin-mutant aortas.

**Figure 5:**
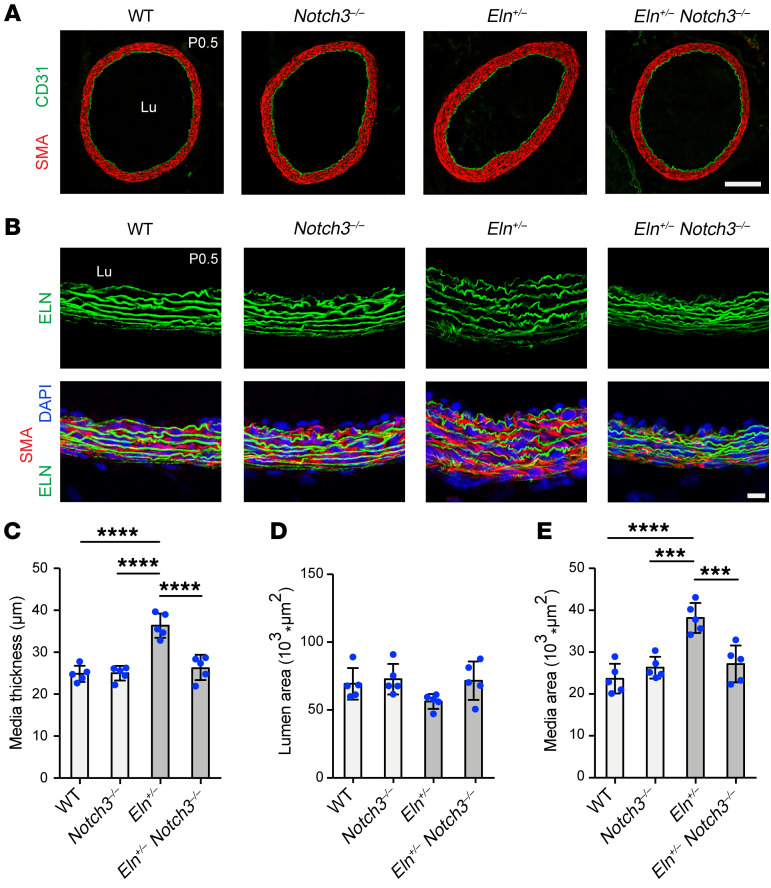
*Notch3* deletion in *Eln^+/–^* mutants reduces aortic muscularization. (**A** and **B**) Transverse sections of the ascending aorta from pups at P0.5 of indicated genotype were stained for SMA and CD31 in **A** and for ELN, SMA, and nuclei (DAPI) in **B**. Lu, lumen. Scale bars: 100 μm (**A**) and 10 μm (**B**). (**C**–**E**) Histograms represent medial thickness (**C**), lumen area (**D**), and medial area (**E**) from **A**. *n =* 5 mice per group. ***P < 0.001; ****P < 0.0001 by 1-way ANOVA with Tukey’s post hoc test. All data are averages ± SD.

**Figure 6:**
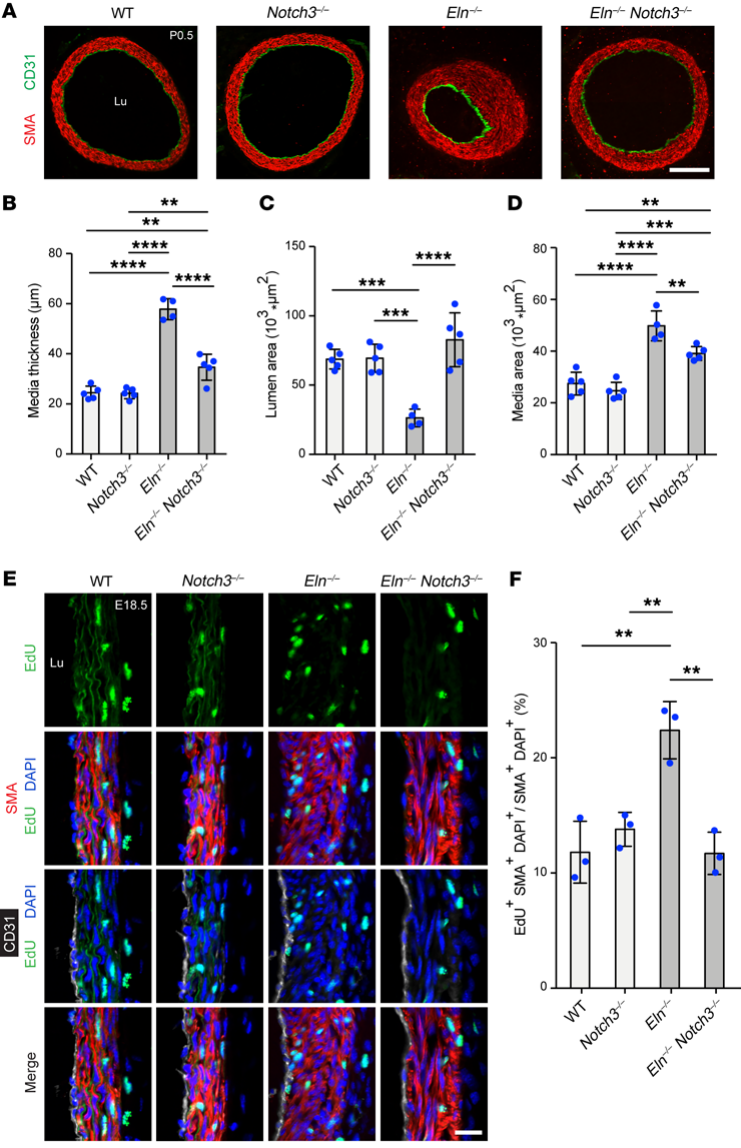
On the *Eln^–/–^* background, *Notch3* deletion attenuates hypermuscularization and stenosis and reduces SMC proliferation. (**A**) Transverse sections of the ascending aorta from pups at P0.5 of indicated genotype were stained for SMA (SMC marker) and CD31 (EC marker). Lu, lumen. Scale bar: 100 μm. (**B**–**D**) Histograms represent medial thickness (**B**), lumen area (**C**), and medial wall area (**D**) of ascending aortas from **A**. *n =* 4 to 5 mice. **P < 0.01; ***P < 0.001; ****P < 0.0001 by 1-way ANOVA with Tukey’s post hoc test. (**E**) EdU was injected in pregnant dams at E18.5, and 8 hours later, embryos of indicated genotypes were collected. Cryosections of ascending aortas were stained for EdU, SMA, CD31, and nuclei (DAPI). Proliferative SMCs were marked by EdU^+^SMA^+^DAPI^+^ cells. Lu, lumen. Scale bar: 25 μm. (**F**) Histogram represents the percentage of SMCs that are proliferative in **E**. *n =* 3 mice. **P < 0.01 by 1-way ANOVA with Tukey’s post hoc test. All data are averages ± SD.

Next, to determine the specific role of NOTCH3 in elastin aortopathy*,* we analyzed the effect of *Notch3* deletion on the *Eln-*mutant background. In neonatal *Eln^+/–^* mice, global deletion of *Notch3* resulted in rescue of medial thickness and medial wall area to near-WT levels (Figure 5). Furthermore, on an elastin-null background, global *Notch3* deletion attenuated excessive muscularization and stenosis of the aorta at P0.5 as compared with mice WT for *Notch3* (Figure 6A). Quantitative analysis revealed that similar to DAPT treatment, compound *Notch3^–/–^* *Eln^–/–^* mutants had a 40% ± 8% reduction in medial thickness, 314% ± 73% increase in lumen area, and 21% ± 5% reduction in medial wall area in comparison with *Eln^–/–^* mice (Figure 6, B–D). Taken together, these findings from pharmacological and genetic inhibition studies indicate that the pathway involving γ-secretase and NOTCH3 plays a key role in the pathogenesis of aortic disease in elastin mutants.

We and others have previously shown that in *Eln^–/–^* aortas, SMCs are hyperproliferative, contributing to increased arterial wall cellularity and stenosis (3, 15, 20). Given that *Notch3* deletion mitigates aortic hypermuscularization and stenosis in *Eln^–/–^* mice (Figure 6, A–D), we next assessed SMC proliferation in aortas of these mice. Dams pregnant with E18.5 embryos were injected with the thymidine analog 5-ethynyl-2′-deoxyuridine (EdU). Eight hours later, embryos were harvested, and transverse cryosections of ascending aortas were stained for EdU, SMA, CD31, and nuclei (DAPI) (Figure 6E). In comparison with WT or *Notch3^–/–^* mice, *Eln^–/–^* mice displayed an approximately 2-fold increase in proliferative aortic SMCs, as marked by EdU, SMA, and DAPI, and this increase was abrogated in compound *Notch3^–/–^* *Eln^–/–^* mice (Figure 6F). A previous study demonstrated that NOTCH3 promotes haSMC survival and proliferation via ERK pathway activation (38). We found that *ELN* knockdown in haSMCs induces ERK phosphorylation (Supplemental Figure 8, A and B) and that reduction of NOTCH3 in haSMCs decreases transcript levels of the prosurvival gene *BIRC5* and proliferation-inducing transcriptional factor *E2F1* (Supplemental Figure 8C). Overall, these data suggest that inhibition of the NOTCH3 pathway during elastin deficiency helps attenuate the aortic phenotype by preventing excessive SMC proliferation.

In addition to SMC hyperproliferation, deficient circumferential growth has been shown to contribute to elastin aortopathy in a model of partial elastin deficiency wherein human elastin is expressed in *Eln^–/–^* mice (44). Our analysis revealed a minor reduction (~10%) in the external diameter of *Eln^–/–^* versus WT aortas, and this reduction was abrogated in compound *Notch3^–/–^* *Eln^–/–^* mice (Supplemental Figure 9). Taken together, these data suggest that deletion of *Notch3* in the *Eln*-null background rescues the aortic phenotype primarily by inhibiting SMC hyperproliferation but also with a minor contribution of improving deficient circumferential growth.

Elastin is critical for lung development, and *Eln^–/–^* mice display dilated distal air sac structures and emphysema (Supplemental Figure 10, A and C), which is likely the major cause of early postnatal death (45, 46). We next assessed whether pharmacological or genetic inhibition of the NOTCH3 pathway could improve lung structure and prolong survival of *Eln^–/–^* mice. Unfortunately, on the *Eln^–/–^* background, DAPT treatment or *Notch3* deletion did not rescue lung phenotype or prolong survival (Supplemental Figure 10).

### Elastin deficiency increases NOTCH3-mediated integrin β3 levels.

**Figure 7:**
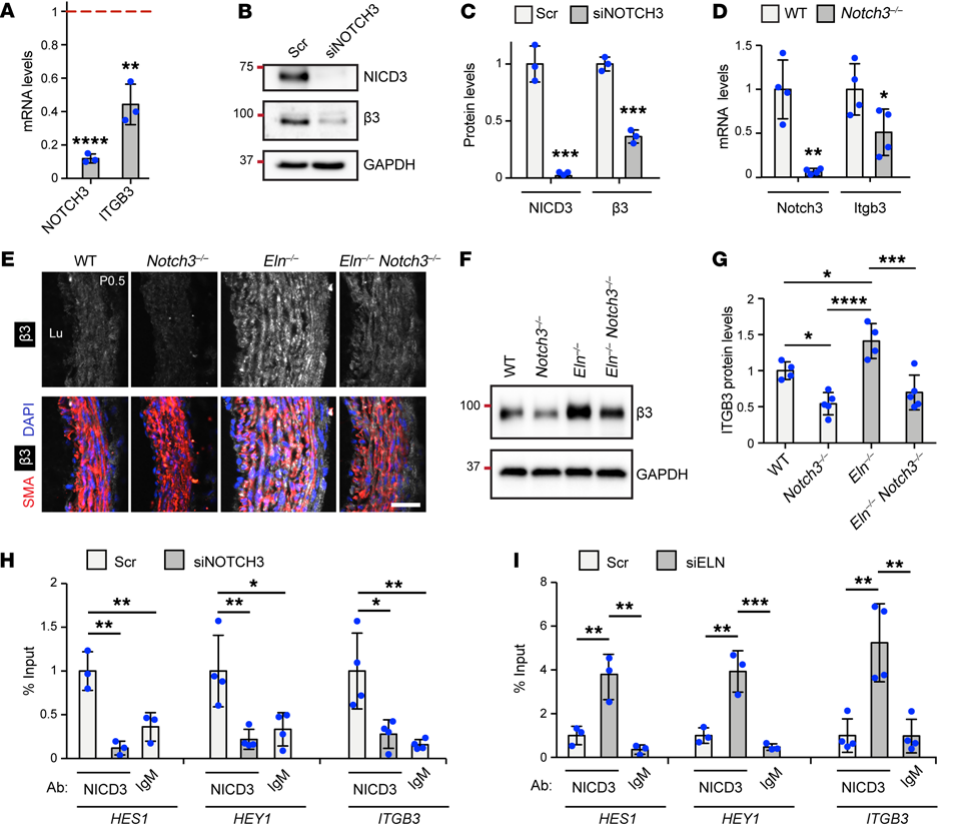
NOTCH3 regulates integrin β3, and elastin silencing promotes NICD3 binding to *ITGB3*. (**A**–**C**) haSMCs were treated with Scr or siNOTCH3 RNA, and then cell lysates were analyzed. In **A**, histogram represents transcript levels of *NOTCH3* and *ITGB3* relative to *18S* rRNA as assessed by qRT-PCR and normalized to Scr treatment (*n =* 3). Western blots for NICD3, integrin β3, and GAPDH are shown in **B**, with densitometry of protein bands relative to GAPDH and normalized to Scr in **C** (*n =* 3). **P < 0.01; ***P < 0.001; ****P < 0.0001 vs. Scr by Student’s *t* test. (**D**) RNA isolated from aortas of WT or *Notch3^–/–^* pups at P0.5 was analyzed by qRT-PCR. Histogram represents mRNA levels of *Notch3* and *Itgb3* relative to *18S* rRNA and normalized to WT (*n =* 4 mice). *P < 0.05, **P < 0.01 vs. WT by Student’s *t* test. (**E**) Ascending aortic transverse sections of indicated genotypes at P0.5 stained for integrin β3, SMA, and nuclei (DAPI) (*n =* 3 mice). Lu, lumen. Scale bar: 25 μm. (**F** and **G**) Aortic lysates from mice of indicated genotype at P0.5 analyzed by Western blotting for integrin β3 and GAPDH in **F**, with densitometry of protein bands relative to GAPDH and normalized to WT in **G** (*n =* 4–5 mice). *P < 0.05; ***P < 0.001; ****P < 0.0001 by 1-way ANOVA with Tukey’s post hoc test. (**H** and **I**) ChIP was performed with antibodies directed against NICD3 or IgM control in haSMCs pretreated with Scr or siNOTCH3 in **H** and Scr or siELN in **I**. qPCR was then conducted with primers specific for regions upstream of the *HES1*, *HEY1*, or *ITGB3* transcription start sites. Graph shows qPCR results calculated by the percentage input method (*n =* 3). *P < 0.05; **P < 0.01; ***P < 0.001 by 1-way ANOVA with Tukey’s post hoc test. All data are averages ± SD.

Our previous studies demonstrated that integrin β3 expression, activation, and signaling are upregulated in the aortic media of *Eln^–/–^* mice and SVAS and WBS patients (3). In elastin mutants, enhanced integrin β3–mediated signaling results in SMC misalignment and hyperproliferation, and pharmacological or genetic inhibition of integrin β3 attenuates aortic hypermuscularization and stenosis (3). Although little is known about the regulation of *ITGB3* transcription, NOTCH3 silencing has previously been shown to reduce integrin β3 levels in cultured cells (47). Herein, we initially confirmed these findings by demonstrating that siRNA-mediated knockdown of NOTCH3 reduces levels of *ITGB3* mRNA by 53% ± 7% and protein by 64% ± 6% in haSMCs (Figure 7, A–C). To extend these findings to the in vivo setting, the aortas of WT and *Notch3*- and/or *Eln*-knockout pups at P0.5 were studied. Similar to results with cultured SMCs, quantitative real-time reverse transcription PCR (qRT-PCR) of isolated aortic RNA revealed an approximately 50% reduction in *Itgb3* transcript levels in *Notch3^–/–^* compared with WT newborns (Figure 7D). In addition, transverse sections of the ascending aorta were stained for integrin β3 (Figure 7E), and aortic lysates were assessed for integrin β3 protein levels by Western blot analysis (Figure 7, F and G). Consistent with our previous investigations (3), integrin β3 staining was upregulated in SMCs of *Eln^–/–^* aorta compared with that of WT. More importantly, our results herein indicate that on the *Eln^–/–^* background, deletion of *Notch3* markedly reduces integrin β3 staining.

To further evaluate NOTCH3-mediated regulation of *ITGB3* expression in elastin aortopathy, we next investigated the hypothesis that NICD3 binds the *ITGB3* gene and that this interaction is enhanced in elastin mutants. haSMCs were treated with Scr or *NOTCH3*-specific siRNA and then subjected to ChIP. Protein-DNA complexes were immunoprecipitated with an antibody directed against NICD3 or an isotype-matched control antibody. Recovered DNA was analyzed by qPCR to assess NICD3 enrichment at the promoters of the *HES1*, *HEY1* (positive controls), and *ITGB3* genes. Our results indicate that NICD3 binds the *ITGB3* proximal promoter region in haSMCs, and this interaction is significantly diminished with NOTCH3 knockdown (Figure 7H). Moreover, in a second set of ChIP experiments, we observed that siELN pretreatment enhances binding of NICD3 to the *HES1*, *HEY1*, and *ITGB3* promoter region (Figure 7I). Overall, these results indicate that in SMCs in culture and in mice, the NOTCH3 pathway induces integrin β3 levels and NICD3 binds to the *ITGB3* promoter, and elastin depletion augments these effects.

### Elastin reduction enhances JAG1 levels in aortic SMCs and ECs.

**Figure 8:**
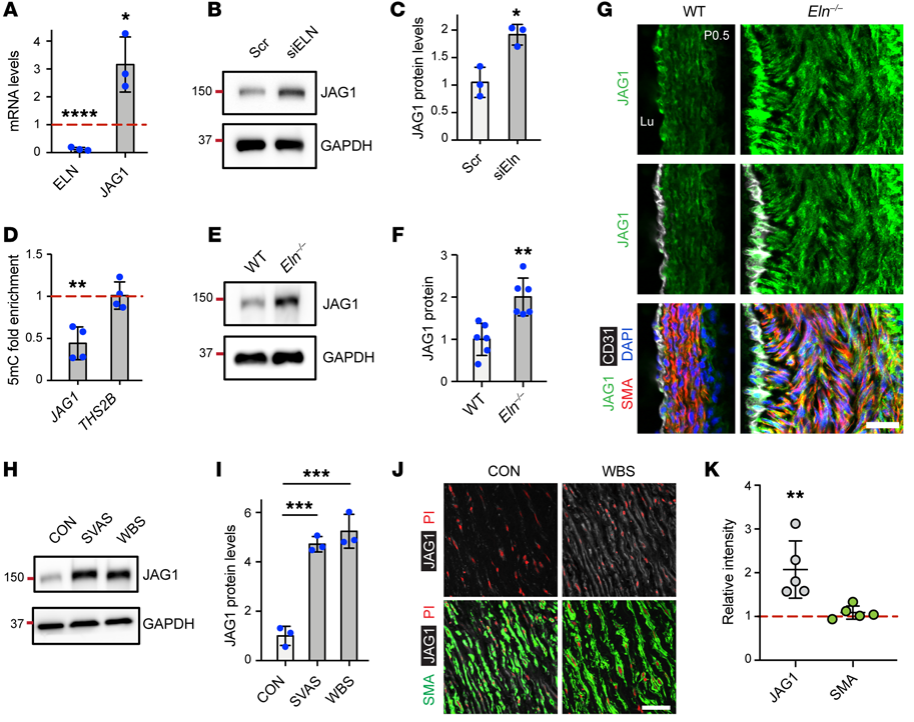
Elastin deficiency in SMCs induces JAG1 upregulation. (**A**–**C**) haSMCs were treated with Scr or siELN RNA, and then lysates were analyzed. In **A**, histogram represents *ELN* and *JAG1* transcript levels relative to *18S* rRNA as assessed by qRT-PCR and normalized to Scr treatment (*n =* 3). Western blots for JAG1 and GAPDH are shown in **B**, with densitometry of protein bands relative to GAPDH and normalized to Scr in **C** (*n =* 3). *P < 0.05, ****P < 0.0001 vs. Scr by Student’s *t* test. (**D**) Methylated DNA (5mC) ChIP from haSMCs pretreated with Scr or siELN. Histogram represents 5mC levels at promoter regions of *JAG1* or *THS2B* (positive control) by qPCR and normalized to Scr (*n =* 4). **P < 0.01 vs. Scr by Student’s *t* test. (**E** and **F**) Aortic lysates from WT or *Eln^–/–^* mice at P0.5 were analyzed by Western blotting for JAG1 and GAPDH (for each blot, 2 aortas were pooled per genotype), with densitometry of JAG1 protein bands relative to GAPDH and normalized to WT (*n =* 6 mice). **P < 0.01 vs. WT by Student’s *t* test. (**G**) Transverse sections of ascending aorta from WT and *Eln^–/–^* mice at P0.5 were stained for JAG1, CD31, SMA, and nuclei (DAPI). *n =* 3 mice. Lu, lumen. Scale bar: 25 μm. (**H** and **I**) Protein levels of JAG1 and GAPDH in iPSC-SMCs derived from control or WBS or SVAS patients as assessed by Western blotting with densitometric analysis of JAG1 normalized to GAPDH (*n =* 3). ***P < 0.001 by 1-way ANOVA with Tukey’s post hoc test. (**J**) Aortic sections from a WBS male patient (46 years old) and control male (53 years old) stained for JAG1, SMA, and nuclei (PI). Scale bar: 50 μm. (**K**) Column scatter plot represents fluorescence intensity of JAG1 and SMA immunostaining in aortic sections of WBS patients (*n =* 5) normalized to age-matched controls (*n =* 11). Intensity was quantified on 8 to 10 microscopic fields per patient. **P < 0.01 vs. control by Student’s *t* test. All data are averages ± SD.

As the NOTCH ligand JAG1 is implicated in early arterial morphogenesis (25–28, 47), we next investigated the role of JAG1 during aortopathy in the context of elastin insufficiency. Elastin silencing in haSMCs resulted in increased JAG1 mRNA and protein levels (Figure 8, A–C). Similarly to the promoter regions of *PSEN1* and *PSEN2*, elastin silencing reduced DNA methylation (5mC) at the *JAG1* promoter, correlating with increased gene expression (Figure 8D). Additionally, lysates of aortas isolated from *Eln^–/–^* pups at P0.5 had upregulated levels of JAG1 protein (Figure 8, E and F). Staining of transverse ascending aortic sections from these pups revealed increased JAG1 levels in the hypermuscularized tunica media (Figure 8G). The relevance of these findings to the human elastin aortopathies, nonsyndromic SVAS and WBS, was evaluated by assessing JAG1 levels in iPSC-SMCs derived from patients with these diseases as well as in the WBS aorta. SVAS and WBS iPSC-SMCs had greater than 4-fold higher JAG1 protein levels as compared with those cells derived from control human iPSC-SMCs (Figure 8, H and I). Similarly, immunostaining of WBS aortas revealed JAG1 upregulation (Figure 8, J and K). Activation by JAG1 is critical in propagating NOTCH activation through developing layers of the arterial media (28). To further evaluate JAG1-mediated downstream signaling, haSMCs were seeded on recombinant JAG1–coated culture dishes. Our results demonstrated that JAG1 stimulation induces *HES1*, *HEY1*, and *JAG1* transcript levels (Supplemental Figure 11).

Interestingly, in addition to SMCs, anti-JAG1 staining was also increased in ECs of aortic sections of *Eln^–/–^* mice at P0.5 (Figure 8G). We postulated that aortic ECs upregulate JAG1 in response to altered ECM lacking elastin, largely derived from SMCs. In large elastic vessels, such as the aorta and pulmonary artery, elastin is predominately produced by SMCs (16, 48, 49), and indeed, our qRT-PCR analysis of cultured cells indicated 300-fold enrichment of *ELN* transcripts in haSMCs compared with haECs (Supplemental Figure 12A). haECs were cultured on ECM derived from haSMCs pretreated with Scr or siELN (Supplemental Figure 12B), and the data indicate that haECs had increased levels of JAG1 transcript (but not other NOTCH ligand transcripts) and protein in response to haSMC-derived elastin-deficient ECM (Supplemental Figure 12, C–E). Collectively, these results indicate that elastin deficiency stimulates vascular cell JAG1 expression in cultured human cells and in vivo in mice and humans.

### Jag1 deletion with Acta2-CreER^T2^, but not Cdh5-Cre, attenuates hypermuscularization and stenosis in elastin mutants.

**Figure 9:**
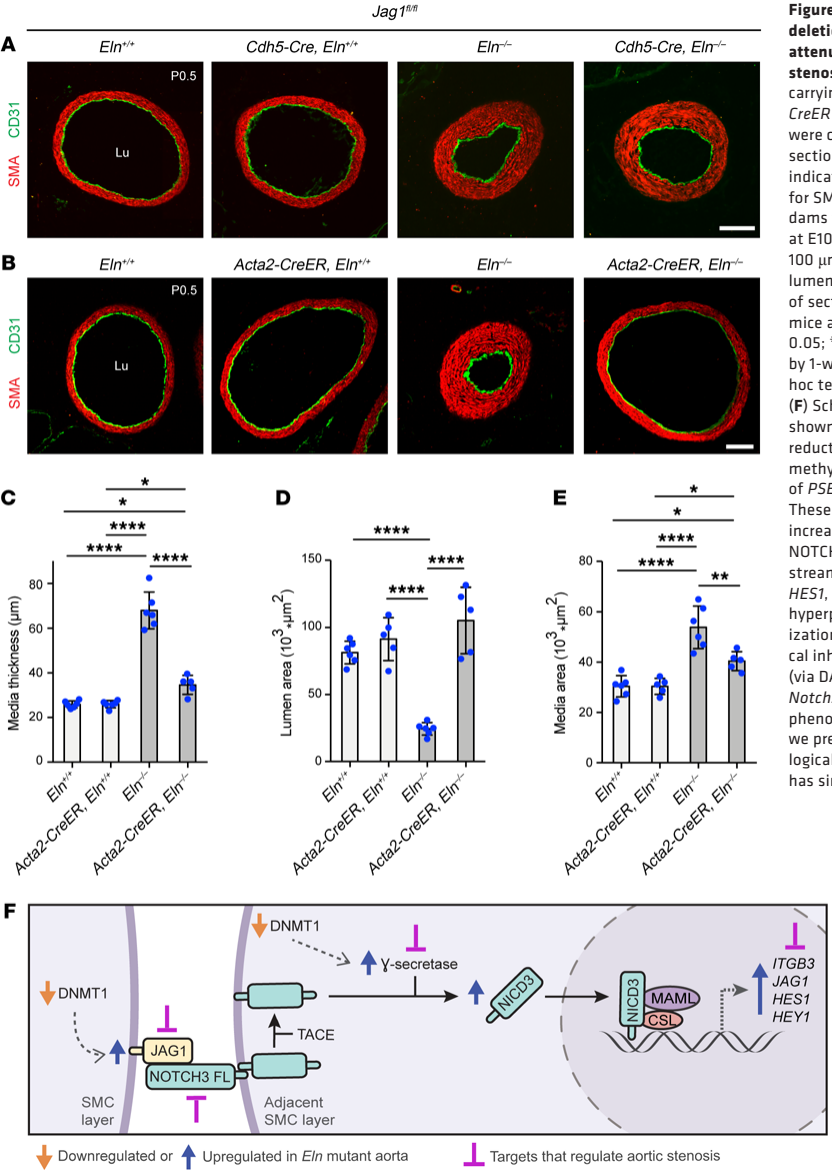
In elastin-mutant mice, deletion of *Jag1* in SMCs but not ECs attenuates muscularization and stenosis. (**A** and **B**) *Jag1^fl/fl^* pups also carrying no Cre, *Cdh5*-*Cre*, or *Acta2-CreER^T2^* and either WT or null for *Eln* were collected at P0.5. Transverse sections of the ascending aorta from indicated genotypes were stained for SMA and CD31. In **B**, pregnant dams were injected with tamoxifen at E10.5. Lu, lumen. Scale bars: 100 μm. (**C**–**E**) Medial thickness (**C**), lumen area (**D**), and medial area (**E**) of sections of ascending aortas from mice as in **B** (*n =* 5 to 6 mice). *P < 0.05; **P < 0.01; ****P < 0.0001 by 1-way ANOVA with Tukey’s post hoc test. Data are averages ± SD. (**F**) Schematic of study findings is shown. During elastin deficiency, reduction in DNMT1-mediated DNA methylation results in expression of *PSEN1*, *PSEN2*, and *JAG1* genes. These gene products culminate in increased NICD3 and activation of NOTCH3 pathway members (downstream genes, including *JAG1*, *ITGB3, HES1*, and *HEY1*), leading to SMC hyperproliferation, hypermuscularization, and stenosis. Pharmacological inhibition of γ-secretase complex (via DAPT) or genetic deletion of *Notch3* or SMC *Jag1* attenuates aortic phenotype of *Eln^–/–^* mice. Note that we previously found that pharmacological or genetic inhibition of *ITGB3* has similar effects (3).

Given the increased JAG1 in vascular cells, we next evaluated the effect of *Jag1*-specific deletion in ECs and SMCs on the hypermuscularization and stenosis phenotype of *Eln*-mutant mice. For investigation of EC JAG1, *Jag1^fl/fl^* pups that were also carrying no Cre or the constitutive *Cdh5-Cre* and either *Eln^+/+^* or *Eln^–/–^* were analyzed (Figure 9A). At P0.5, newborns were genotyped, and the ascending aortas were sectioned transversely and stained for CD31 and SMA. The increased medial thickness and area and reduced lumen area in *Jag1^fl/fl^* pups of the *Eln^–/–^* background were not altered by the presence of *Cdh5*-*Cre* (Supplemental Figure 13, A–C) despite very high (97% ± 2%) deletion efficiency of *Jag1* in ECs as assessed by qRT-PCR (Supplemental Figure 13D), suggesting that EC JAG1 is not requisite for elastin aortopathy. The aorta of *Cdh5*-*Cre* *Jag1^fl/fl^* adults has previously been shown to have some acellular gaps in the subendothelial SMC layer (47). Consistent with this prior study, our results demonstrated rare gaps in the inner layer of the tunica media of *Cdh5*-*Cre* *Jag1^fl/fl^* aortas at P0.5; however, at this same time point, the aortas of *Eln^–/–^* *Cdh5*-*Cre* *Jag1^fl/fl^* mice had markedly more acellular gaps, which were located throughout the media (Supplemental Figure 14).

To determine the role of JAG1 in SMCs during elastin aortopathy, pups with conditional SMC-specific *Jag1* deletion on an *Eln*-WT or -null background were generated (Figure 9B). We injected pregnant dams at E10.5 with 1 mg of tamoxifen and concomitant 0.25 mg of progesterone to minimize the incidence of dystocia (3), and a *Jag1* deletion efficiency of 68% ± 14% was achieved in aortic SMCs of newborn pups (Supplemental Figure 15). At P0.5, transverse ascending aortic sections were stained for CD31 and SMA (Figure 9B). In comparison with controls, the medial thickness and wall area of *Jag1^fl/fl^* *Eln^–/–^* newborns were increased and the lumen area was decreased; these changes were prevented in *Acta2-CreER^T^* *Jag1^fl/fl^* *Eln^–/–^* pups (Figure 9, C–E). Thus, SMC deletion of *Jag1* attenuates hypermuscularization and stenosis in elastin mutants. Taken in their entirety, our findings identify the NOTCH pathway and specifically, JAG1, NOTCH3, and γ-secretase as key molecular players in the pathogenesis of elastin aortopathy and also as promising therapeutic targets for the human diseases SVAS and WBS (Figure 9F).

## Discussion

Obstructive arterial diseases, including atherosclerosis, restenosis, pulmonary hypertension, and the genetic elastin arteriopathy SVAS, are characterized by elastic fiber deficiency, degradation, and/or fragmentation, and excess SMCs. The cellular and molecular mechanisms linking elastin defects and hypermuscularization remain incompletely understood, which is a major obstacle to the development of novel effective therapies. Indeed, major vascular surgery is the only therapy for the arterial obstruction of SVAS and carries a sizable morbidity and mortality risk. There is a dire need for intense investigation into mechanisms underlying the pathogenesis of elastin aortopathy, as insights from these studies have far-reaching impact on diverse vasculoproliferative diseases.

Although no prior investigations to our knowledge have evaluated the function of the NOTCH signaling pathway during elastin insufficiency, NOTCH plays a myriad of critical roles during vascular development. Indeed, mice bearing deletion of diverse NOTCH pathway components have severe cardiovascular defects, many of which result in embryonic lethality (22, 24, 50). Directly relevant to the current study, the NOTCH pathway regulates arterial SMC differentiation (25, 26, 28, 31). Furthermore, mutations in NOTCH pathway components in humans cause cardiovascular disorders, such as CADASIL, bicuspid aortic valve disease, and Alagille syndrome (30, 51–53). On the other hand, inhibition of the NOTCH pathway has been implicated as a potential therapeutic strategy for select vascular diseases, including pulmonary hypertension, tumor angiogenesis, and pathological vascular permeability in diabetic retinopathy (29, 50, 54).

In the current study, we utilize a wide array of elastin deficiency models — knockdown in cultured human vascular cells, genetic and pharmacological inhibition in mouse models, and iPSC-SMCs and aortic samples from nonsyndromic SVAS and/or WBS patients — to demonstrate that the JAG1/NOTCH3/γ-secretase pathway is overactive in SMCs with elastin depletion (Figure 1). The catalytic component of the enzyme γ-secretase, PSEN1 or -2, cleaves type 1 transmembrane proteins, such as amyloid precursor protein and NOTCH receptors (55), and interestingly, our data demonstrate that elastin deficiency increases SMC levels of the γ-secretase complex, including PSEN1 and -2 (Figure 2). These results reveal that perturbation of γ-secretase in SMCs plays an important role in elastinopathy.

Epigenetic modifications influence gene expression by altering chromatin accessibility and play a central role in regulating SMC behavior during physiological and pathological conditions, including several cardiovascular diseases (33). However, epigenetic regulation in the context of elastin deficiency is previously unexplored, and our data implicate elastin deficiency in modulating the epigenetic landscape of SMCs (Figure 3). Specifically, loss of elastin reduces DNMT1, a pivotal epigenetic regulatory enzyme that catalyzes DNA methylation to induce gene silencing (41). Upon loss of elastin, levels of global as well as locus-specific 5mC mark at the promoters of *PSEN1*, -*2*, and *JAG1* are decreased. These data shed light on molecular mechanisms underlying JAG1/NOTCH3 pathway activation in elastin deficiency. The global hypomethylation of elastin-deficient SMCs is intriguing. One possible explanation is that extensive ECM remodeling associated with elastin deficiency might directly or indirectly regulate DNMT1 levels and trigger genome-wide hypomethylation. In addition to expression levels, the enzymatic activity of DNMTs may be altered in *Eln^–/–^* SMCs. Future investigations into how elastin deficiency regulates DNMT1 and the SMC epigenome promise to reveal further insights into the biology of elastin and SMCs.

Pharmacological inhibition of the γ-secretase complex in *Eln^–/–^* mice reduces the aortic hypermuscular and stenosis phenotype (Figure 4). As described previously (15, 21), the aortas of *Eln^+/–^* mice display thinner and additional elastic lamellar units with excess SMC layers (Supplemental Figures 6 and 7). Importantly, from a clinical standpoint, our results demonstrate that postnatal treatment with a γ-secretase inhibitor substantially reverses hypermuscularization in *Eln^+/–^* mice (Supplemental Figure 7). In addition, genetic deletion of *Notch3* significantly reduces aortic stenosis in *Eln^–/–^* mice and hypermuscularization in both *Eln^+/–^* and *Eln^–/–^* mice (Figures 5 and 6).

Elastin is a critical component of diverse organ systems and is indispensable for lung development. *Eln^–/–^* mice have dilated distal air sacs in the lung and die in the immediate postnatal period (20, 45, 46). NOTCH3 inhibition in *Eln^–/–^* mice neither attenuates lung developmental defects nor prolongs survival (Supplemental Figure 10), indicating that, not surprisingly, inhibiting the NOTCH3 pathway is insufficient to entirely overcome the massive burden of total elastin loss. In contrast to *Eln*-null mice, *Eln^+/–^* mice display normal lung development (56). Thus, targeting the NOTCH3 pathway may be a promising therapeutic strategy for human aortic elastinopathies (i.e., nonsyndromic SVAS and WBS), which are caused by *ELN* haploinsufficiency and generally lack lung parenchymal disease. Because pathological complications of SVAS manifest from infancy onward and often worsen with time (sometimes resulting in sudden death), therapeutic intervention should be considered soon after diagnosis (57, 58).

Elastin is a potent regulator of SMC behavior. Tropoelastin inhibits cultured *Eln^–/–^* aortic SMC proliferation and migration, and an elastin matrix sheath coating of metal stents reduces balloon-overexpansion-induced neointimal SMC accumulation and arterial obstruction in porcine coronary arteries (7). Conversely, *Eln^–/–^* aortas accumulate excess SMCs in the subendothelial region of the vessel wall (3, 20), but mechanisms underlying this hypermuscularization are incompletely understood. Herein, our findings suggest that the NOTCH3 pathway regulates SMC proliferation in *Eln^–/–^* aortas, as compound *Notch3^–/–^* *Eln^–/–^* mutants display reduced aortic SMC proliferation relative to *Eln^–/–^* and comparable proliferation to WT mice (Figure 6). It has been previously shown that NOTCH2 and NOTCH3 have opposing effects on SMCs; NOTCH2 inhibits proliferation by reducing MAP kinase activity, whereas NOTCH3 promotes cell survival and proliferation via MAP kinase induction (38). In addition, NOTCH3 induces expression of prosurvival genes and prevents apoptosis, while NOTCH2 does not alter apoptosis (38). In agreement with these studies, herein we find that loss of elastin in SMCs upregulates MAP kinase activity (Supplemental Figure 8, A and B) and does not alter NICD2 levels (Supplemental Figure 2) but enhances NICD3 (Figure 1). Consistent with prior studies (38), our data indicate that expression levels of the prosurvival gene *BIRC5* and proliferative gene *E2F1* are reduced by NOTCH3 silencing (Supplemental Figure 8C). Thus, the cumulative effect of elastin deficiency on levels of NOTCH3 (increased) and NOTCH2 (unchanged) may be critical in promoting excessive SMC proliferation and potentially in altering cell survival. Additionally, previous investigations of partial elastin deficiency in which *Eln^–/–^* mice express human elastin showed that impaired outward growth contributes to aortic stenosis (44). Our data suggest that reduced outward growth has a small contribution to the development of stenosis in *Eln^–/–^* model and *Notch3* deletion in the elastin-null background improves the circumferential growth (Supplemental Figure 9).

We previously reported that integrin β3 expression, activation, and signaling are upregulated in SMCs of elastin-deficient mice and inhibition of integrin β3 attenuates aortic hypermuscularization and stenosis in these mice (3). Although integrin β3 is widely studied, relatively little is known about regulation of *Itgb3* transcription. Herein, our initial studies in this area confirmed prior work (47) that knockdown of NOTCH3 in haSMCs attenuates integrin β3 transcript and protein levels, and we extended this work by showing similar effects in the aorta of *Notch3*-null mice (Figure 7). Furthermore, our data indicate that in *Eln*-null mice, *Notch3* deletion attenuates integrin β3 expression. Most interestingly, ChIP studies demonstrate that NICD3 binds the *Itgb3* promoter in haSMCs, and elastin deficiency enhances this interaction.

In addition to the role of the NOTCH3 in elastin aortopathy, we focused on the ligand JAG1, which has been implicated as an important player in arterial wall morphogenesis (25–28, 47). Our results indicate increased expression of JAG1 in aortic SMCs with elastin deficiency in cell culture, in mice, and in WBS patients as well as in SVAS and WBS iPSC-SMCs (Figure 8). Mechanistically, elastin deficiency mediates hypomethylation of the *JAG1* promoter, resulting in gene activation (Figure 8D). Culturing SMCs on JAG1-coated dishes results in robust induction of JAG1 and downstream effectors HES1 and HEY1 (Supplemental Figure 11). These results suggest that exposure to JAG1 can mediate rapid activation of the JAG1/NOTCH pathway positive feed-forward loop in haSMCs (27). Immunohistochemical analysis also revealed enhanced expression of JAG1 in the EC layer of the aorta in *Eln^–/–^* as compared with WT mice. The initial study describing *Eln^–/–^* mice reported a lack of evidence for altered ECs (20). More recently, Wagenseil and colleagues demonstrated that the hypermuscular ascending aorta of *Tagln*-*Cre* *Eln^fl/fl^* mice lacks intact elastic fibers in the media and has a disrupted internal elastic lamellae (that separates ECs from SMCs), whereas EC-specific deletion of *Eln* does not alter aortic histology (16). Consistent with these results, we find that *ELN* transcript levels are highly enriched (>300-fold) in haSMCs as compared with haECs in culture, and interestingly, ECM produced by elastin-silenced SMCs is sufficient to induce EC JAG1 expression (Supplemental Figure 12). Collectively, our data indicate that JAG1 levels are induced in both ECs and SMCs during elastin deficiency.

Prior studies have demonstrated a key role of EC JAG1 in aortic SMC development and/or maintenance (26, 47). *Cdh5*-*Cre* *Jag1^fl/fl^* mice are viable, with sporadic acellular gaps in the inner layer of the tunica media (47). Our results indicate that *Eln^–/–^* *Cdh5*-*Cre* *Jag1^fl/fl^* pups at P0.5 have numerous acellular gaps throughout the aortic tunica media (Supplemental Figure 14); however, this EC loss does not attenuate the stenotic phenotype (Figure 9 and Supplemental Figure 13). Finally, in contrast to *Cdh5*-*Cre*–induced EC deletion, SMC-specific deletion of *Jag1* with *Acta2-CreER^T2^* attenuates aortic hypermuscularization and stenosis in *Eln^–/–^* neonates (Figure 9). It has previously been shown that *Jag1* deletion with the constitutive *Tagln*-*Cre* results in patent ductus arteriosus, defective descending aorta SMC differentiation, and early postnatal lethality but does not alter ascending aorta morphogenesis (25). Similarly, we do not observe phenotypic changes in the ascending aorta with SMC-specific deletion of *Jag1* (utilizing *Acta2-CreER^T2^*) in elastin-WT mice; however, in the *Eln^–/–^* background, there is a significant reduction in aortic hypermuscularization. These results highlight the critical role of JAG1 in SMC expansion during elastin aortopathy.

Taken together, our results show that elastin deficiency in SMCs results in reduction of DNMT1 levels and DNA methylation, which induces expression of key NOTCH pathway genes (*JAG1*, *PSEN1*, and *PSEN2*) and triggers NOTCH3 activation. Elastin-deficient SMCs display increased levels of (a) the NOTCH ligand JAG1; (b) γ-secretase complex, including PSEN1 and -2; (c) NICD3; (d) NOTCH pathway downstream effectors, including integrin β3 and the HES/HEY family of transcription factors; and (e) ERK activation, which culminate in excess SMC proliferation and accumulation and stenosis of the ascending aorta (Figure 9F). Additionally, NOTCH3 regulates the prosurvival gene *BIRC5* and proliferative factor E2F1, which may provide a survival advantage to elastin-deficient SMCs. Our findings reveal critical mechanistic insights into arterial hypermuscularization during elastin insufficiency and identify the JAG1/NOTCH3/γ-secretase pathway as a key mediator of elastin aortopathy. These studies suggest that inhibiting specific components of this pathway in SMCs is a promising therapeutic strategy for human diseases SVAS and WBS and potentially other obstructive arterial diseases associated with elastin insufficiency and excess SMCs.

## Methods

Further information can be found in Supplemental Methods.

### Animal studies and treatments.

C57BL/6 WT, B6 129S1-Notch3tm1Grid/J (*Notch3^+/–^*; ref. 59), and *Jag1^tm2Grid^* (*Jag1^fl/fl^*; ref. 60) mice were from The Jackson Laboratory. *Eln^tm1Dyl^* (*Eln^+/−^*; ref. 20); Tg(Cdh5-cre)7Mlia (*Cdh5*-*Cre*; ref. 61); and Tg(Acta2-cre/ERT2)51Pcn (*Acta2-CreER^T2^*; ref. 62) mice have been previously described. All mice were maintained on the C57BL/6 background. Mice were bred and embryos or pups were harvested at different ages, with E0.5 considered the time of vaginal plug. All agents were injected intraperitoneally. EdU (Invitrogen, 10 mg/kg body weight) was administered to pregnant dams on E18.5 and embryos were collected 8 hours later. Tamoxifen (Sigma-Aldrich, 1 mg) with concomitant progesterone (Sigma-Aldrich, 0.25 mg) was administered on E10.5, and pups were analyzed immediately after birth at P0.5. Pregnant dams were injected with 1 mg DAPT (Calbiochem) or vehicle (4% DMSO in corn oil) on E14.5 and E15.5, and pups were collected at P0.5. For postnatal analysis, WT or *Eln^+/−^* mice were injected daily with DAPT (1.5 mg/kg body weight) from P2.5 to P5.5, and pups were collected at P7.5.

### Immunohistochemistry.

After euthanasia, embryos or pups were fixed in 4% paraformaldehyde for 2 hours, transferred to 30% sucrose in PBS, embedded in OCT compound (Tissue-Tek), frozen, and stored at –80°C. Transverse serial cryosections of ascending aortas of 10 μm thickness were cut starting immediately caudal to the aortic arch and continuing for 200 μm inferiorly, and sections near the aortic arch were utilized for analyses (3–4 sections per mouse). Cryosections were incubated with blocking solution (5% goat serum, 0.1% Triton X-100 in PBS) and then with primary antibodies diluted in blocking solution overnight at 4°C. On the next day, sections were washed with 0.5% Tween 20 in PBS (PBS-T) and incubated with secondary antibodies diluted in blocking solution for 1 hour. Primary antibodies used were rat anti-CD31 (BD Pharmigen, 553370; 1:100), rabbit anti-JAG1 (Abcam, ab7771; 1:100), rabbit anti-PSEN1 (Cell Signaling Technology, 5643; 1:100), rabbit anti-PSEN2 (Cell Signaling Technology, 9979; 1:100), rabbit anti–integrin β3 (Abcam, ab210515; 1:100), anti-HES1 (Cell Signaling Technology, 11988; 1:1000), and directly conjugated FITC or Cy3 anti-SMA (Sigma-Aldrich, C6198; 1:300). Secondary antibodies were conjugated to Alexa Fluor 488, 555, or 647 (Molecular Probes) or Dylight 555 or 649 (Jackson ImmunoResearch) fluorophores and used at 1:500 dilution. DAPI (Sigma-Aldrich, D9542; 1:500) was used for nuclear staining. CF633 hydrazide was used for elastin staining (Sigma-Aldrich, SCJ4600037; 1:1000). A Click-iT EdU Alexa Fluor 594 Imaging kit (Invitrogen) was used to assess proliferation. For detecting DNA methylation, cryosections were incubated in 2N HCl for 45 minutes at 37°C and washed with 0.1 M boric acid prior to incubation in blocking solution for 1 hour. Sections were then stained for 5mC (Cell Signaling Technology, 28692; 1:100) overnight at 4°C followed by secondary antibody staining as described above. The percentages of medial SMCs with the 5mC mark were scored.

Aortas from patients with WBS (*n =* 5; refs. 3, 9, 63, 64) and human controls (*n =* 11) (as described in Supplemental Table 1) were fixed in formalin, paraffin embedded, and sectioned. Paraffin was removed from sections of human aortas with Histo-Clear (National Diagnostics), and after ethanol washes, sections were rehydrated into water. Rehydrated sections were incubated in boiling antigen retrieval buffer (Dako) for 20 minutes. Sections were allowed to cool at room temperature for 1 hour and then rinsed twice in PBS-T and blocked for 1 hour in 5% goat serum, 0.5% Triton X-100 in PBS. Slides were incubated overnight with anti-JAG1 antibody conjugated to Alexa Fluor 647 (Santa Cruz Biotechnology, sc390177; 1:50) at 4°C. The next day, sections were washed with PBS-T and incubated for 1 hour with FITC directly conjugated anti-SMA antibody (Sigma-Aldrich, F3777; 1:500). Propidium iodide (Sigma-Aldrich, P4170; 1:500) was used for nuclear staining.

### Quantification of staining intensity and parameters of aortic morphology.

Quantifications used ImageJ software (NIH). Fluorescence intensity of immunostaining for JAG1 and SMA on formalin-fixed, paraffin-embedded human aortic sections (10 μm thick) was measured from 5 WBS patients and 11 controls (6–8 fields per sample). Measurements from aortas of WBS patients were normalized to that of age-matched controls as detailed in Supplemental Table 1. The number of SMC nuclei per high-power field did not appear to differ substantively between WBS and control groups stratified by age (Supplemental Figure 16). The medial wall thickness from transverse sections of murine aorta were calculated by measuring the distance between the inner aspect of the inner and the outer aspect of the outer SMA^+^ medial layers (8 measurements per section and 3–4 sections per mouse). Medial and lumen areas were calculated by measuring the area of SMA staining and the area interior to CD31 staining, respectively (3–4 sections per mouse). The external diameter was measured on aortic transverse sections as the length of a line having endpoints at the outer aspects of the outermost SMA^+^ medial layer and passing through the center of the lumen (measurements of 2 perpendicular lines per section and 3–4 sections per mouse). All morphometric analyses of the aorta were done in a blinded fashion. One potential limitation is that because perfusion fixation was not performed in vivo, lumen loss measured on sections could be an artifact of postmortem vessel constriction.

### Patient-derived iPSC-SMC generation.

Human undifferentiated iPSCs reprogrammed from skin fibroblasts of a WBS patient (WS1-iPSC line C; refs. 65, 66), an *ELN*-mutant nonsyndromic SVAS patient (ELN1; ref. 66), and a control human (CT2; ref. 66) were used to generate iPSC-SMC progenitor cell lines via an embryoid body stage (65, 66). The age and sex of iPSC donors are provided in Supplemental Table 2. As described previously (67), the SMC progenitor cells were expanded in Matrigel-coated 6-well plates in smooth muscle growth medium (Medium 231, growth supplement) (Life Technologies). Cells were passaged when they reached 80% to 90% confluence. To induce differentiation, the iPSC-SMC progenitors were trypsinized and plated in smooth muscle differentiation medium (Medium 231, differentiation supplement) (Life Technologies) on 0.1% gelatin–coated 6-well plates for 6 days. RNA was isolated before and after SMC differentiation and utilized for qRT-PCR analysis of SMC markers. Protein lysates from the differentiated iPSC-SMCs were collected and utilized for Western blotting.

### Cell culture and siRNA-mediated knockdown.

haSMCs (Lonza) or haECs (ScienCell) were cultured up to passage 6 in M199 medium supplemented with 10% FBS, EGF, and FGF (PromoCell) or complete EC medium (ScienCell), respectively. For gene silencing, siRNA was transfected as described previously (68). Briefly, haSMCs were transfected with Lipofectamine 2000 (Life Technologies) containing siRNA targeted against *ELN* (Dharmacon, 50 nM) or *NOTCH3* (Origene, 50 nM), or Scr RNA for 6 hours. Cells were then washed in M199 and cultured for 72 hours prior to collection for qRT-PCR or Western blot analysis.

### qRT-PCR.

For cultured haSMCs or haECs, RNA was isolated with the RNeasy Plus Kit (Life Technologies). For newborn mice, PBS was perfused through the left ventricle, the entire aorta from the root to the iliac arteries was dissected, and aortic RNA was extracted with mechanical homogenization in TRIzol (Invitrogen) and PureLink RNA columns (Invitrogen). The isolated RNA was reverse transcribed with the iScript cDNA Synthesis Kit (Bio-Rad), and qRT-PCR was performed on a CFX96 Real-Time System (Bio-Rad) using SsoFast EvaGreen supermix (Bio-Rad) and primer pairs as per Supplemental Table 3. Normalized mRNA levels are relative to *18S* rRNA for cultured cells and to *18S* rRNA or *Gapdh* for murine aortas.

### Western blot.

Lysates of cultured cells were prepared by solubilizing cells in 1.5× Laemmli sample buffer at 95°C for 10 minutes. Lysates from aortas were prepared by pooling aortas from 2 pups for each genotype per sample and mechanically lysing in 1.5× Laemmli sample buffer on ice with a glass pestle tissue homogenizer (Pyrex). Aortic lysates were then centrifuged at 16,000*g* and 4°C for 2 minutes, supernatants were collected, and protein concentration was determined by BCA assay (Thermo Fisher Scientific). Protein samples from cultured cells or aortas were resolved by 7%–15% SDS-PAGE, transferred to Immobilon PVDF membranes (Millipore), blocked with 5% nonfat dry milk or bovine serum albumin, washed in TBS-T, and probed with primary antibodies overnight at 4°C. Membranes were incubated with HRP-conjugated secondary antibodies (Dako), washed in TBS-T, developed with Supersignal West Pico Maximum Sensitivity Substrate (Pierce), and analyzed with the G:BOX imaging system (Syngene). Primary antibodies used for Western blot analysis were rabbit anti-NOTCH3 (Cell Signaling Technology, 2889; 1:500), rabbit anti-NOTCH2 (Cell Signaling Technology, 5732; 1:500), rabbit anti-DNMT1 (Cell Signaling Technology, 5032; 1:1000), rabbit anti-JAG1 (Cell Signaling Technology, 70109; 1:1000), rabbit anti-HES1 (Cell Signaling Technology, 11988; 1:1000), rabbit anti-NCT (Cell Signaling Technology, 5665; 1:1000), rabbit anti-PSEN1 (Cell Signaling Technology, 5643; 1:1000), rabbit anti-PSEN2 (Cell Signaling Technology, 9979; 1:1000), rabbit anti-PEN2 (Cell Signaling Technology, 8598; 1:1000), rabbit anti–integrin β3 (Abcam, 197662; 1:1000), rabbit anti–p-ERK (Cell Signaling Technology, 9101; 1:1000), rabbit anti-ERK (Cell Signaling Technology, 9102; 1:1000), rabbit anti-GAPDH (Cell Signaling Technology, 2218; 1:2500), rabbit anti–human ELN (generated against human aortic α elastin; 1:500), and rabbit anti–mouse ELN (raised against exons 6–17 of recombinant mouse tropoelastin; 1:500) (14, 69).

### 5mC ChIP.

Methylated-DNA immunoprecipitation (MeDIP) was carried out using a previously published protocol (70). Briefly, enrichment of methylated DNA was performed using the MeDIP kit (Diagenode, C02010021) as per the manufacturer’s instructions. A total of 1 μg of genomic DNA was isolated from haSMCs pretreated with Scr or siELN and sheared to 200 to 500 bp using the Covaris sonicator. Methylated DNA was captured by incubation with anti-5mC monoclonal antibody coupled to magnetic beads overnight at 4°C. The beads were washed and methylated DNA was eluted. The recovered DNA and input fractions were analyzed by qPCR to assess enrichment of the methylated DNA at the *PSEN1*, *PSEN2*, and *JAG1* gene promoters.

The following forward and reverse primer pairs spanning canonical CSL binding motifs upstream of the transcription start site were used: *PSEN1* 5′-GTTCTCCCCGCAATCGTTTC-3′ and 5′-CACCGTTGTCGTCATTTCCG; *PSEN2* 5′-CCCCAGTGGACGAGGGAAC-3′ and 5′- CTCCAGCGGAGTTTACGCA-3′; *JAG1* 5′-GTAGAAGAACCAGGGCCCCA-3′ and 5′- AGCAACGATCCCTTCCAAGT-3′. The location of the PCR amplification product with *PSEN1* primers is chr14:73,136,336–73,136,451, with *PSEN2* primers is chr1:226,870,512–226,870,633, and with *JAG1* primers is chr20:10,673,792–10,674,673. All reactions were performed in at least triplicate from 4 independent experiments, and data were calculated by the percentage input method. The primer pairs for the *THS2B* promoter (positive control with constitutive 5mC mark) were included in the MeDIP kit.

### Imaging.

Fluorescence images of aortic sections were acquired with a confocal microscope (PerkinElmer UltraView Vox Spinning Disc). Brightfield images of H&E staining were captured using inverted microscopes (Eclipse 80i and Eclipse TS100, Nikon). Volocity software (PerkinElmer) and Adobe Photoshop were used to process images.

### Data and materials availability.

Upon reasonable request, iPSC-derived SMC progenitor lines are available from JE and SM and anti-elastin antibodies are available from RM. All other materials reported in this manuscript are commercially available. All the data are available in the main text and supplementary information.

### Statistics.

A 2-tailed Student’s *t* test and multifactor ANOVA with Tukey’s post hoc test were used to analyze the data after testing for normality and equal variance using GraphPad Prism (version 6.03). Statistical significance threshold was set at a *P* value of less than 0.05. All data are presented as mean ± SD.

### Study approval.

All procedures involving human aorta tissue isolated at the time of surgery or autopsy were approved by the Institutional Review Boards of Yale University (no. 2000020632), Stanford University (no. 12726), University of Pittsburgh (no. PRO10020125), and the New England Organ Bank and complied with all relevant ethical regulations. For patient iPSC generation, all procedures were approved by the Institutional Review Board of the Hospital for Sick Children in Toronto, Canada (no. 1000011232) and the Stem Cell Oversight Committee of the Canadian Institutes of Health Research. All participants, parents or legal guardians provided written informed consent to participate in the study. All mouse experiments were approved by the Institutional Animal Care and Use Committee at Yale University and in accordance with the NIH *Guide for the Care and Use of Laboratory Animals* (National Academies Press, 2011).

## Author contributions

JMD, RC, AN, FZS, ZF, AM, KAM, and DMG conceived of and designed experiments and JMD, RC, AN, JS, and FZS performed them. CK, JE, and SM provided SMC progenitors derived from human iPSCs. RKR, ZU, and GT provided human aortic samples. RM provided the anti-elastin antibodies. JMD and DMG analyzed the results, prepared the figures, and wrote the manuscript. All authors reviewed and provided input on the manuscript.

## Supplementary Material

Supplemental data

## Version 1 01/06/2022

In-Press Preview

## Version 2 03/01/2022

Electronic publication
