# Supplementary material for: JAGGED1/NOTCH3 activation promotes aortic hypermuscularization and stenosis in elastin deficiency
Source: J Clin Invest. 2022 Mar 1;132(5):e142338. doi: 10.1172/JCI142338 (PMC8884911; doi:10.1172/JCI142338)
Supplement: Supplemental data [file jci-132-142338-s199.pdf]

SUPPLEMENTAL INFORMATION

JAGGED1/NOTCH3 activation promotes aortic hypermuscularization and stenosis in elastin deficiency

Jui M. Dave, Raja Chakraborty, Aglaia Ntokou, Junichi Saito, Fatima Z. Saddouk, Zhonghui Feng, Ashish Misra, George Tellides, Robert K. Riemer, Zsolt Urban, Caroline Kinnear, James Ellis, Seema Mital, Robert Mecham, Kathleen A. Martin, Daniel M. Greif

List of Supplemental Items:

- Supplemental Figures and Legends 1-16
- Supplemental Tables 1-3
- Supplemental Methods
- Supplemental References

SUPPLEMENTAL FIGURES

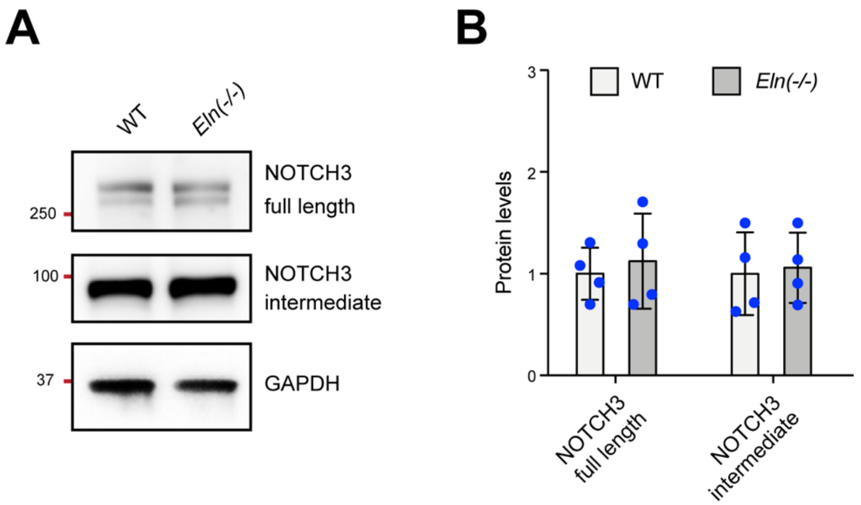

Supplemental Figure 1. Protein levels of NOTCH3 full length and intermediate forms are not changed in *Eln*$^{(-/-)}$ mice. (A) Aortic lysates from wild type (WT) or *Eln*$^{(-/-)}$ mice at P0.5 were subjected to Western blot analysis of NOTCH3 full length, intermediate form and GAPDH. **(B)** Densitometry of protein bands relative to GAPDH and normalized to WT. n=4 mice per group. All data are averages ± SD.

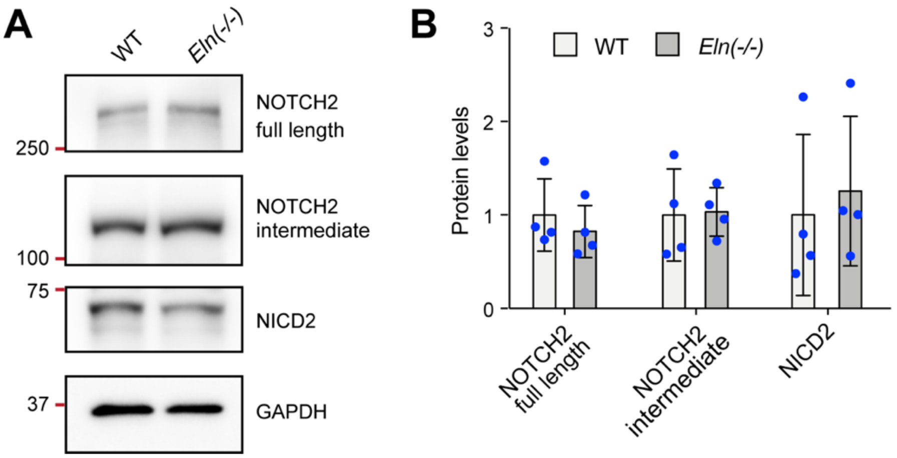

Supplemental Figure 2. NOTCH2 protein levels are not changed in *Eln*$^{(-/-)}$ mice. (A) Aortic lysates from wild type (WT) or *Eln*$^{(-/-)}$ mice at P0.5 were subjected to Western blots for NOTCH2 full-length, NICD2 and GAPDH. **(B)** Densitometry of protein bands relative to GAPDH and normalized to WT. n=4 mice per group. All data are averages ± SD.

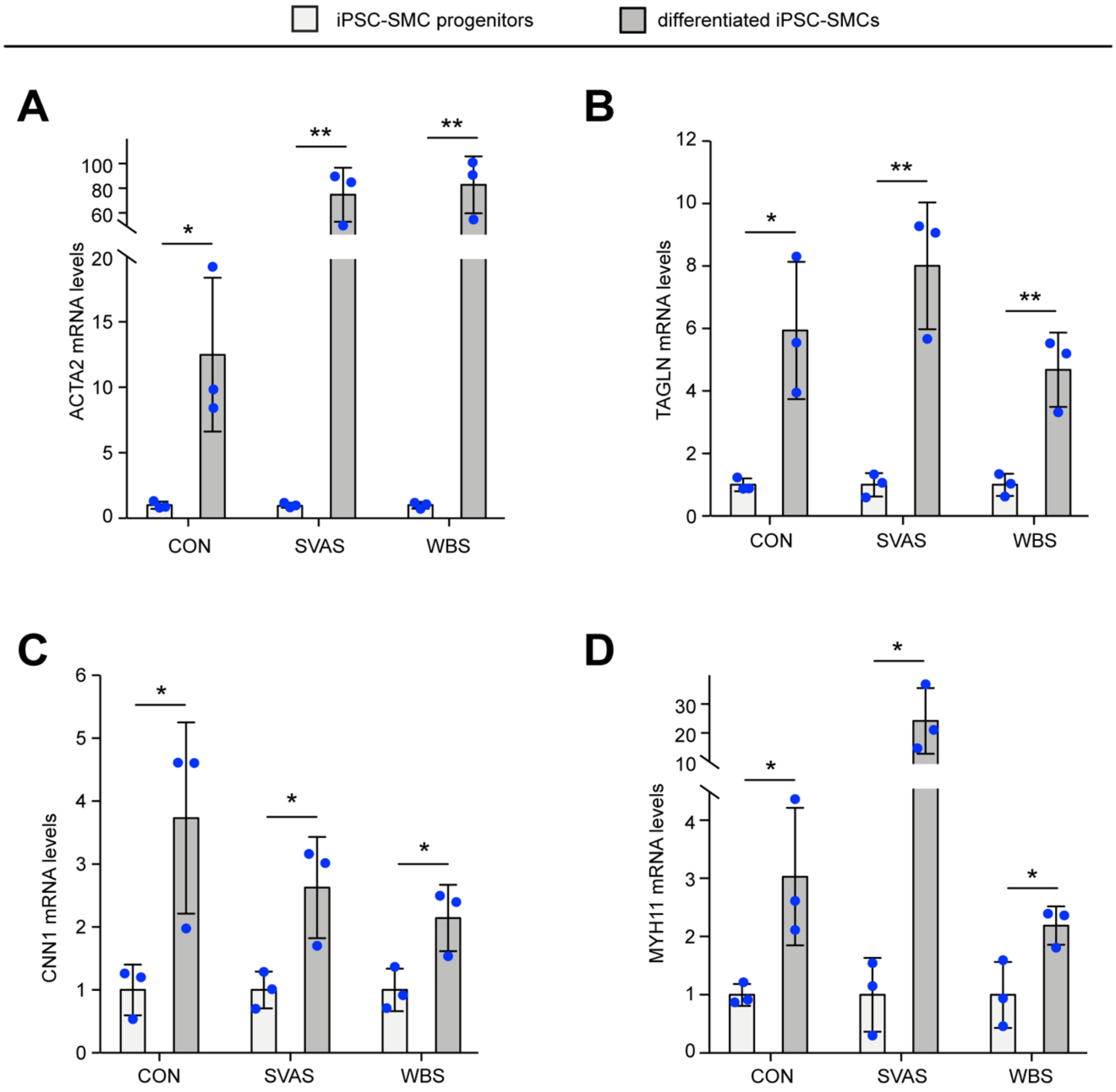

Supplemental Figure 3. SMC marker expression in differentiated human iPSC-SMCs. (A-D) Human iPSC-SMC progenitors from control (CON), SVAS or WBS patients were differentiated into SMCs. Cell lysates were collected before and after differentiation and analyzed. Histograms represent mRNA levels of SMC markers; ACTA2 (A), TAGLN (B), CNN1 (C) and MYH11 (D) relative to 18S rRNA and normalized to iPSC-SMC progenitors (n=3). Student's *t*-test, **p*<0.05, ***p*<0.01 vs. iPSC-SMC progenitors. All data are averages ± SD.

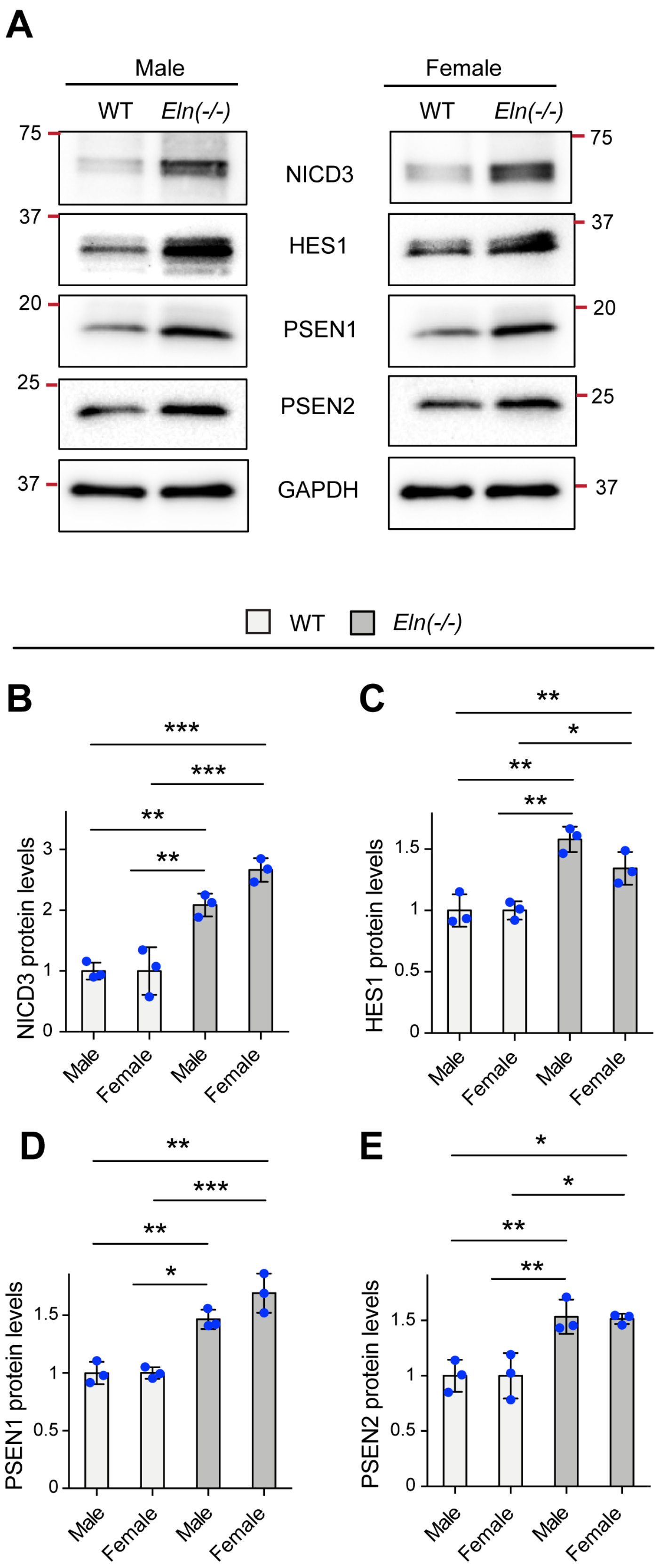
A
Male
Female
WT
Eln(-/-)
NICD3
HES1
PSEN1
PSEN2
GAPDH
75
37
20
25
37
WT
Eln(-/-)
B
NICD3 protein levels
C
HES1 protein levels
D
PSEN1 protein levels
E
PSEN2 protein levels
Male
Female
Male
Female

Supplemental Figure 4. Levels of key NOTCH pathway members in *Eln*$^{(-/-)}$ aorta are not affected by sex. (A) Aortic lysates from WT or *Eln*$^{(-/-)}$ mice from indicated sex at P0.5 were subjected to Western blots for NICD3, HES1, PSEN1, PSEN2 and GAPDH. **(B-F)** Densitometry of protein bands relative to GAPDH and normalized to WT. n=3 mice per group. One-way ANOVA with Tukey's *post hoc* test, *$p<0.05$, **$p<0.01$, ***$p<0.001$. All data are averages ± SD.

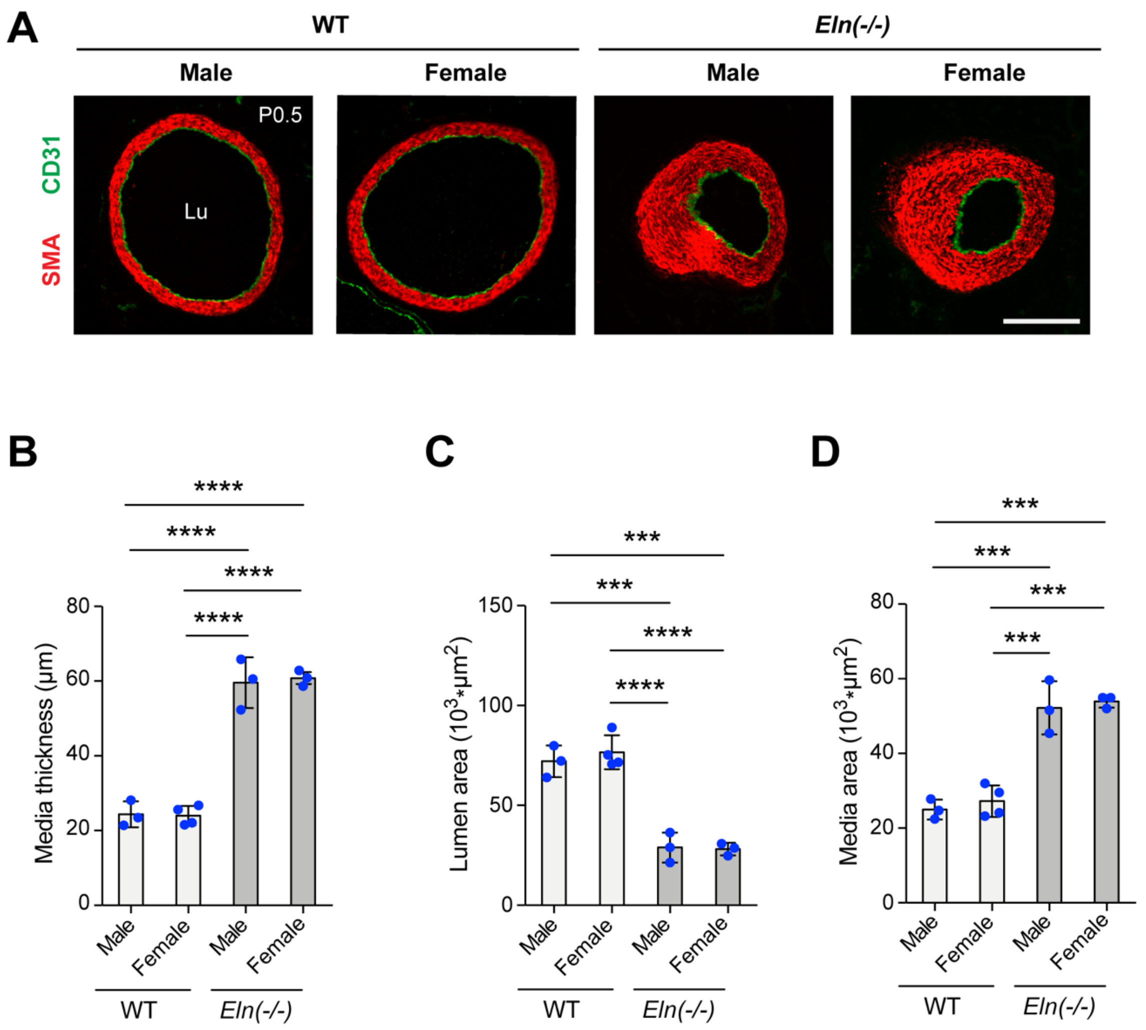

Supplemental Figure 5. Elastin aortopathy development in *Eln*$^{(-/-)}$ mutants is not affected by sex. (A) Transverse sections of the ascending aorta from pups at P0.5 of indicated sex and genotype were stained for SMA and CD31. Lu, lumen. Scale bar, 100 μm. **(B-D)** Histograms represent medial thickness (B), lumen area (C) and medial area (D) of ascending aortas from (A). n=3 mice per group. Two-way ANOVA with Tukey's post hoc test, ***p<0.001, ****p<0.0001. All data are averages ± SD.

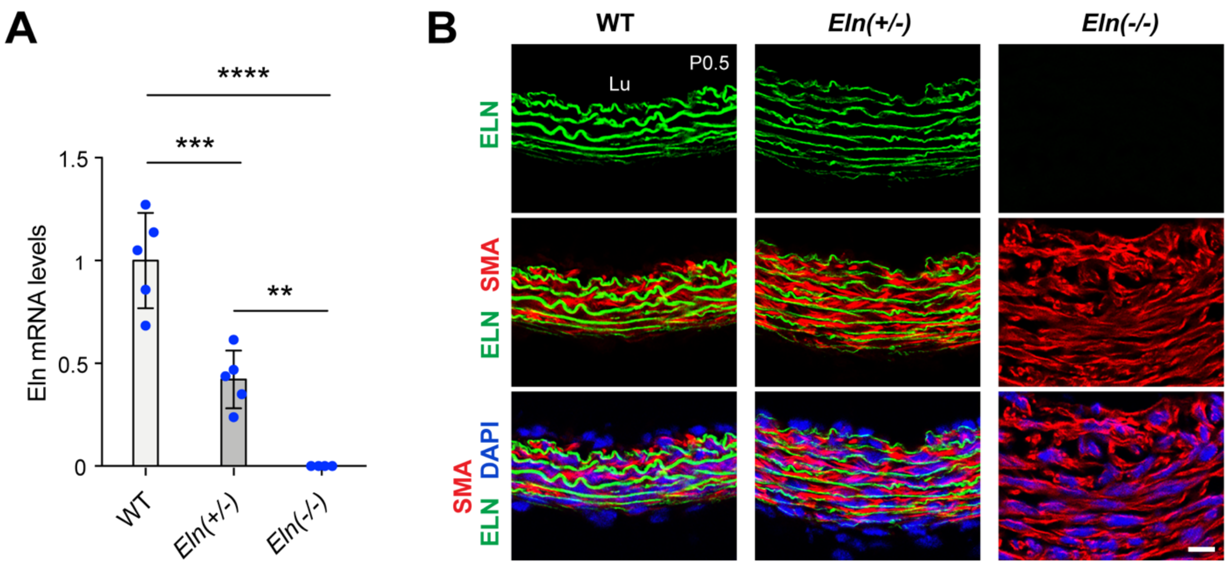

Supplemental Figure 6. Analysis of elastin transcript levels in mice. (A) RNA isolated from aortas of wild type (WT), *Eln*$^{(+/-)}$ or *Eln*$^{(-/-)}$ pups at P0.5 was subjected to qRT-PCR. Histogram represents mRNA levels of Eln relative to 18S rRNA and normalized to WT. n=4-5 mice per group. One-way ANOVA with Tukey's post hoc test, ***p*<0.01. ****p*<0.001, *****p*<0.0001. All data are averages ± SD. **(B)** Transverse sections of the ascending aorta from WT, *Eln*$^{(+/-)}$ or *Eln*$^{(-/-)}$ pups at P0.5 stained for ELN, SMA and nuclei (DAPI). Lu, lumen. Scale bars, 10 µm.

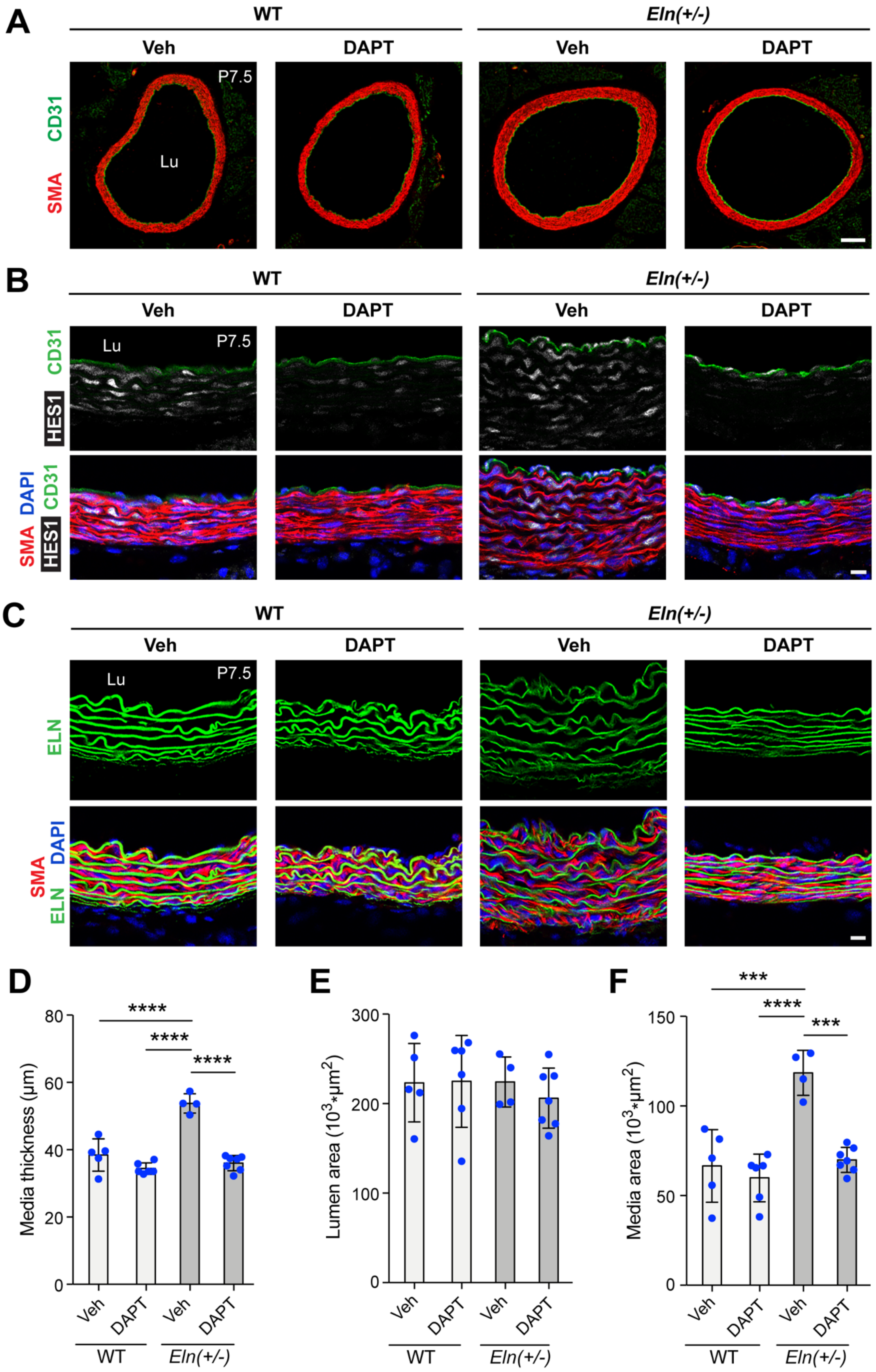
A
WT
Eln(+/-)
Veh
DAPT
Veh
DAPT
CD31
SMA
P7.5
Lu
B
WT
Eln(+/-)
Veh
DAPT
Veh
DAPT
CD31
HES1
DAPI
CD31
SMA
HES1
Lu
P7.5
C
WT
Eln(+/-)
Veh
DAPT
Veh
DAPT
ELN
DAPI
SMA
ELN
Lu
P7.5
D
Media thickness (µm)
0
20
40
60
80
****
****
****
Veh
DAPT
Veh
DAPT
WT
Eln(+/-)
E
Lumen area (10^3*µm^2)
0
100
200
300
Veh
DAPT
Veh
DAPT
WT
Eln(+/-)
F
Media area (10^3*µm^2)
0
50
100
150
***
****
***
Veh
DAPT
Veh
DAPT
WT
Eln(+/-)

Supplemental Figure 7. DAPT treatment mitigates pre-established aortic muscularization. WT or *Eln*$^{(+/-)}$ pups were injected daily with DAPT (1.5mg/kg body weight) from P2.5 to P5.5, and collected at P7.5. **(A)** Transverse sections of the ascending aorta from pups at P7.5 of indicated genotype and treatment were stained for SMA and CD31. **(B, C)** Aortic sections from pups as in (A) were stained for HES1, SMA, CD31 and nuclei (DAPI) in (B) or ELN, SMA and nuclei (DAPI) in (C). Lu, lumen. Scale bars, 100 µm in (A), 10 µm in (B) and (C). **(D-F)** Histograms represent medial thickness (D), lumen area (E) and medial area (F) from (A). n=4-7 mice per group. Two-way ANOVA with Tukey's *post hoc* test, *****p*<0.0001. All data are averages ± SD.

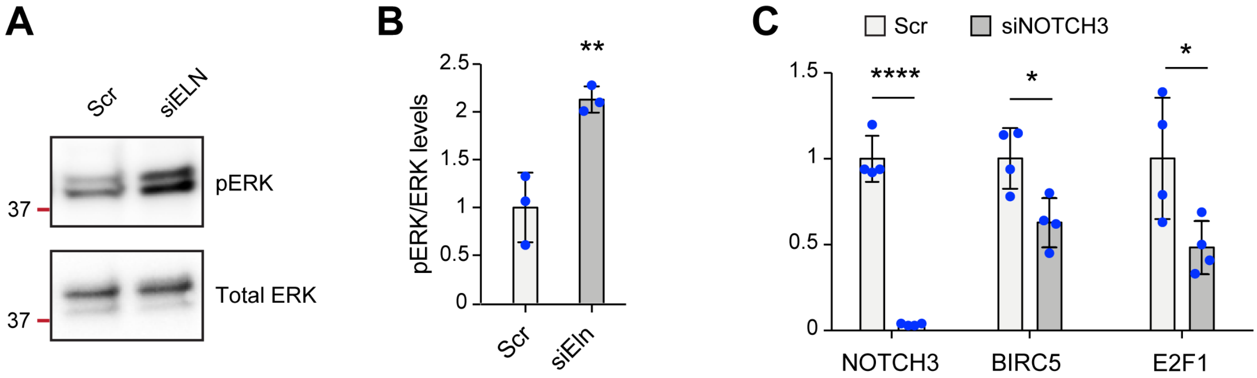

Supplemental Figure 8. NOTCH3 promotes BIRC5 and E2F1 expression and elastin silencing induces pERK activation in haSMCs. (A, B) haSMCs were treated with Scr or siELN RNA, and then protein lysates were analyzed. Western blots for pERK and total ERK are shown in (A) with densitometry of protein bands relative to total ERK and normalized to Scr in (B). n=3. Student's *t*-test, ***p*<0.01 vs. Scr. **(C)** haSMCs were treated with Scr or siNOTCH3 RNA and then lysates were analyzed. Histogram represents NOTCH3, BIRC5 and E2F1 transcript levels relative to 18S rRNA as assessed by qRT-PCR and normalized to Scr treatment (n=4). Student's *t*-test, **p*<0.05, *****p*<0.0001 vs. Scr. All data are averages ± SD.

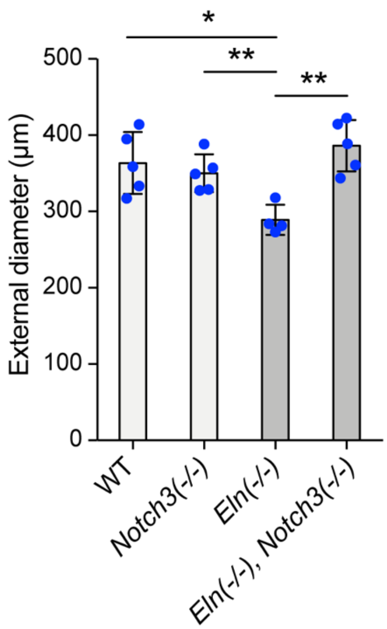

Supplemental Figure 9. Deletion of *Notch3* increases external diameter of the aorta in *Eln*$^{(-/-)}$ mice at P0.5. Histogram represents external diameter of the ascending aorta from indicated genotypes at P0.5 with representative images shown in Figure 5A. n=4-5 mice. One-way ANOVA with Tukey's *post hoc* test, *p<0.05, **p<0.01. Data are averages ± SD.

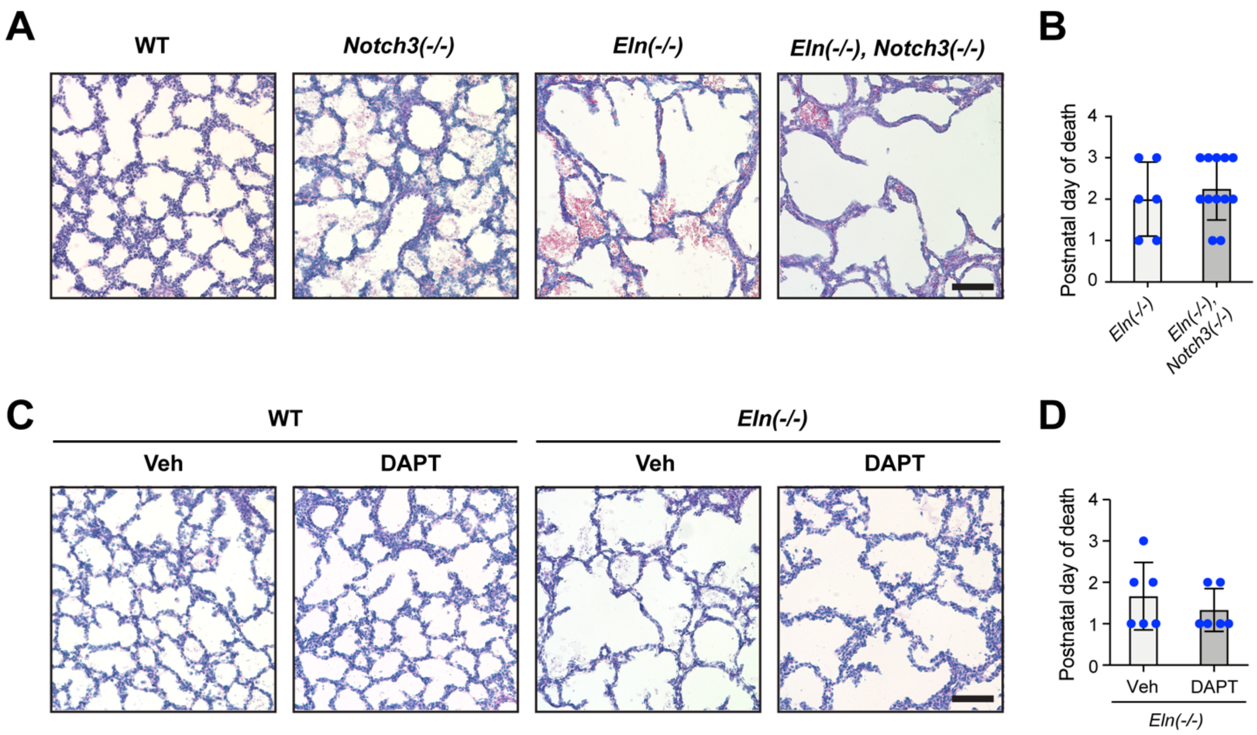

Supplemental Figure 10. ***Notch3*** **deletion or DAPT treatment does not improve survival or lung development in** ***Eln***$^{(-/-)}$ **animals. (A, C)** Lung transverse sections from indicated genotype and/or treatment at P0.5 were subjected to H&E staining. Scale bars, 100 µm. **(B, D)** Histograms represent postnatal day of death in *Eln*$^{(-/-)}$ newborn mice bearing either *Notch3*$^{(-/-)}$ in (B) or treated with vehicle or DAPT in (D), with bars indicating the mean age of death. n=6 *Eln*$^{(-/-)}$ mice and n=12 *Eln*$^{(-/-),}$ *Notch3*$^{(-/-)}$ mice in (B); n=6 *Eln*$^{(-/-)}$ mice treated with vehicle or DAPT in (D). All data are averages ± SD.

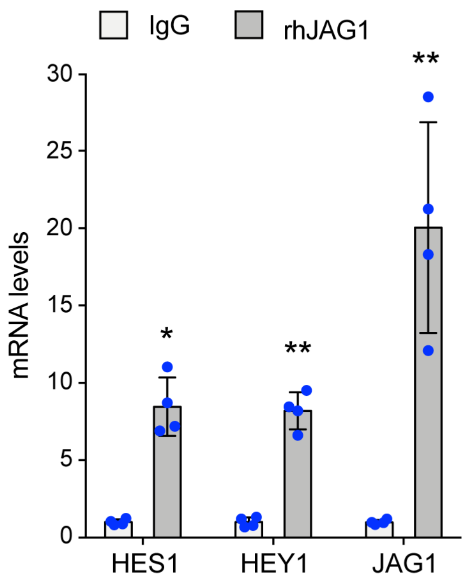

Supplemental Figure 11. rhJAG1 stimulation in haSMCs induces NOTCH pathway and JAG1 expression. haSMCs were seeded on culture plates coated with rhJAG1 or IgG for 24 hours, and lysates were analyzed. Histogram represents transcript levels of HES1, HEY1 and JAG1 relative to 18S rRNA as assessed by qRT-PCR and normalized to IgG treatment (n=4). Student's *t*-test, *$p<0.05$, **$p<0.01$ vs. IgG. All data are averages ± SD.

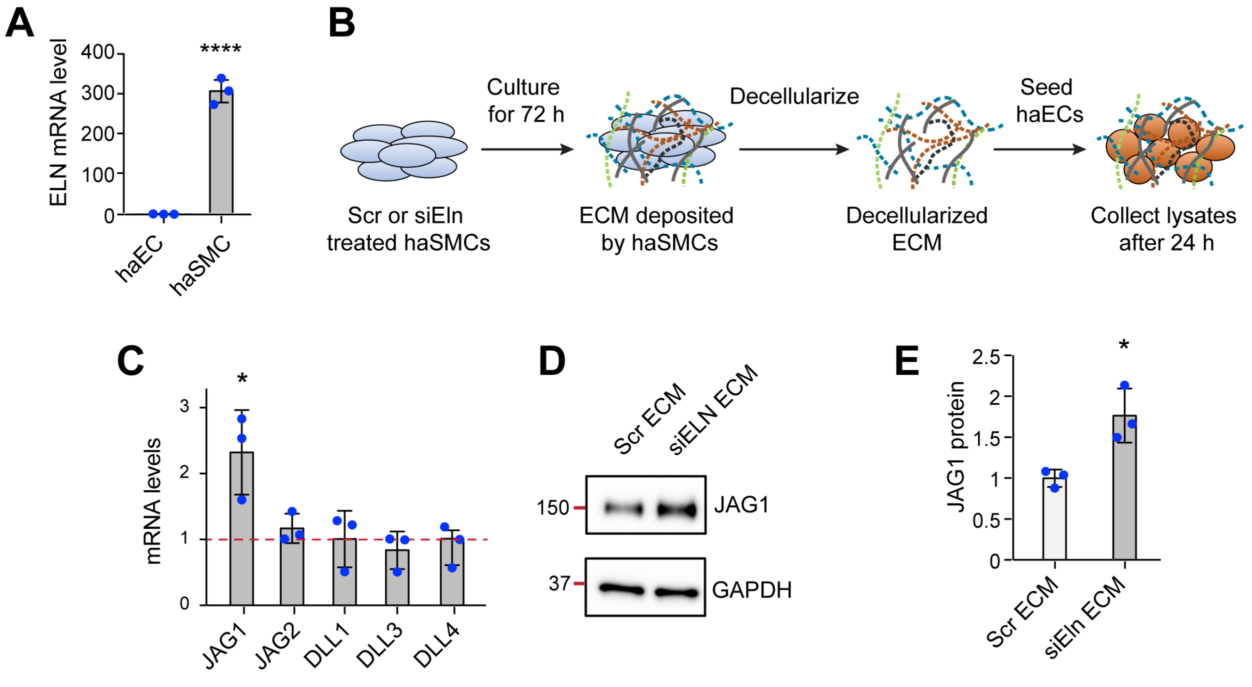

Supplemental Figure 12. Eln mRNA level is much higher in SMCs than ECs, and ECM from elastin deficient SMCs induces EC JAG1. (A) In human aortic ECs (haECs) and SMCs (haSMCs), ELN mRNA levels was assessed by qRT-PCR relative to 18S rRNA and normalized to haSMCs (n=3). Student's *t*-test, ****p<0.0001 vs. haECs. **(B)** Schematic depicting decellularization strategy. haSMCs pre-treated with Scr or siELN were plated for 72 hours and then decellularized to obtain SMC-derived ECM. haECs were cultured on decellularized ECM for 24 hours, and then lysates were collected and analyzed. **(C)** In lysates described in (B), transcript levels of indicated NOTCH ligands relative to 18S rRNA as assessed by qRT-PCR and normalized to Scr SMC-derived ECM (Scr ECM) (n=3). Student's *t*-test, *p<0.05 vs. Scr ECM. **(D, E)** As described in (B), Western blots of JAG1 and GAPDH from haECs seeded on Scr or siELN-SMC derived ECM with densitometry of JAG1 relative to GAPDH and normalized to Scr SMC-derived ECM (n=3). Student's *t*-test, *p<0.05 vs. Scr ECM. All data are averages ± SD.

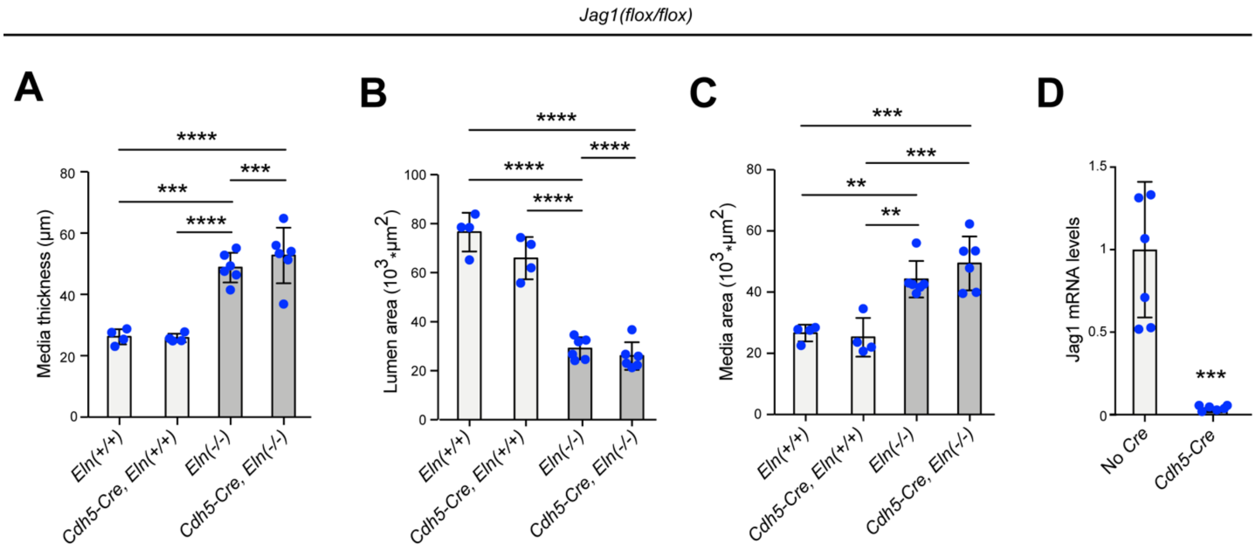

Supplemental Figure 13. *Cdh5-Cre* efficiently deletes *Jag1* in ECs, and elastin aortopathy does not differ between *Jag1$^{(flox/flox)}$*, *Eln$^{(-/-)}$* mice carrying no Cre or *Cdh5-Cre*. (A-C) At P0.5, *Jag1$^{(flox/flox)}$* pups also carrying no Cre or *Cdh5-Cre* and either *Eln$^{(+/+)}$* or *Eln$^{(-/-)}$* were genotyped, and transverse sections of the ascending aorta were stained for SMA and CD31 with representative images shown in Figure 8A. Quantification of medial thickness (A), lumen area (B) and medial area (C) from these sections are shown. n=4-6 mice per each genotype. One-way ANOVA with Tukey's *post hoc* test, ****p<0.0001. **(D)** Lung ECs were isolated from *Jag1$^{(flox/flox)}$* pups also carrying no Cre or *Cdh5-Cre* at P0.5. Histogram depicts levels of Jag1 transcript relative to 18S rRNA in lung EC lysates as assessed by qRT-PCR and normalized to no Cre, *Jag1$^{(flox/flox)}$* mice. n=6 mice per group. Student's t-test, ***p<0.001 vs. *Jag1$^{(flox/flox)}$*. Data are averages ± SD.

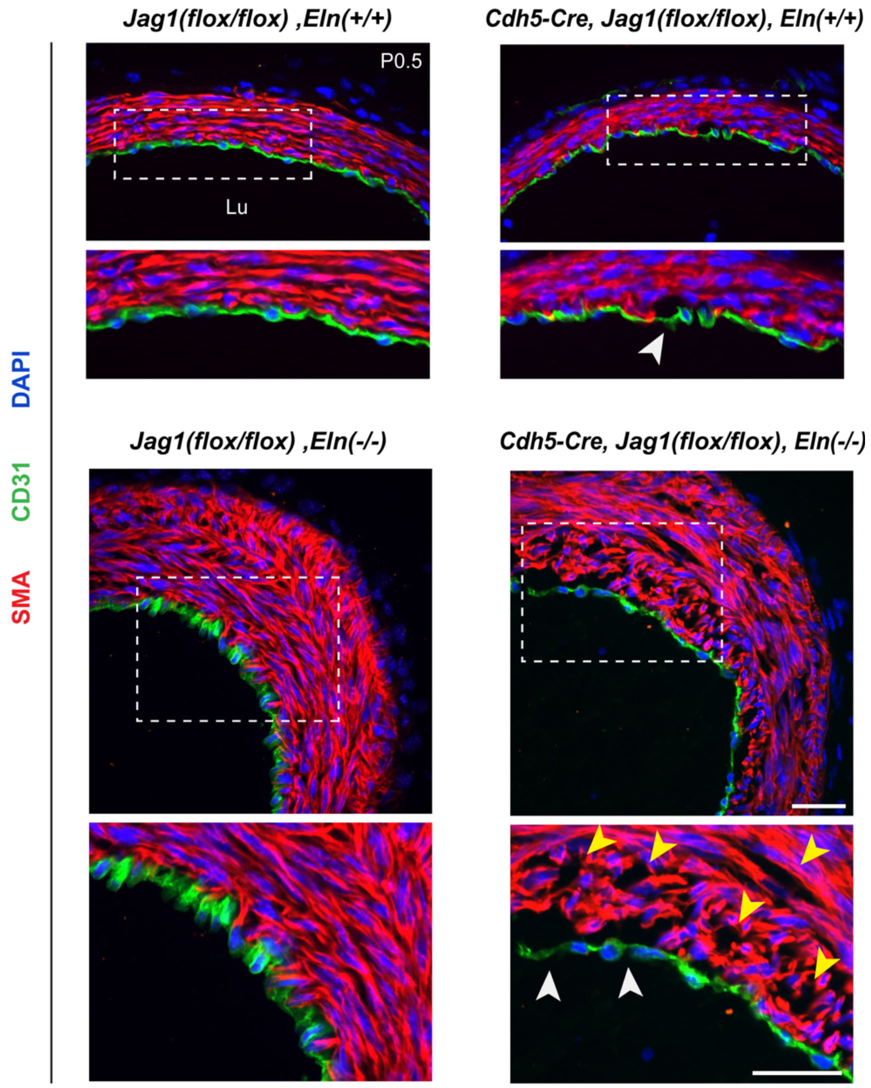

Supplemental Figure 14. EC-specific *Jag1* deletion in *Eln*$^{(-/-)}$ mice induces acellular gaps in the tunica media of the aorta. *Jag1*$^{(flox/flox)}$ pups also carrying no Cre or *Cdh5-Cre* and either wild type or null for *Eln* were collected at P0.5. Transverse sections of the ascending aorta of the indicated genotypes were stained for SMA, CD31 and nuclei (DAPI). For each genotype, close-up images of the boxed areas are shown below. Yellow and white arrowheads indicate acellular gaps in the sub-endothelial or deeper regions of the media, respectively. Lu, lumen. Scale bar, 25 µm

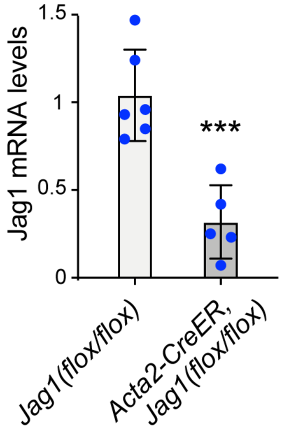

Supplemental Figure 15. *Jag1* deletion efficiency in aortic SMCs of *Acta2-CreER^{T2}*, *Jag$^{(flox/flox)}$* mice. Dams pregnant with *Jag1$^{(flox/flox)}$* embryos also carrying no Cre or *Acta2-CreER^{T2}* were injected with tamoxifen at E10.5. Aortas were isolated from pups at P0.5 and subjected to lysis after adventitia removal. Histogram represents Jag1 transcript levels relative to 18S rRNA as assessed by qRT-PCR and normalized to no Cre, *Jag1$^{(flox/flox)}$* mice. n=5-6 mice per group. Student's *t*-test, ***p<0.001 vs. *Jag1$^{(flox/flox)}$*. Data are averages ± SD.

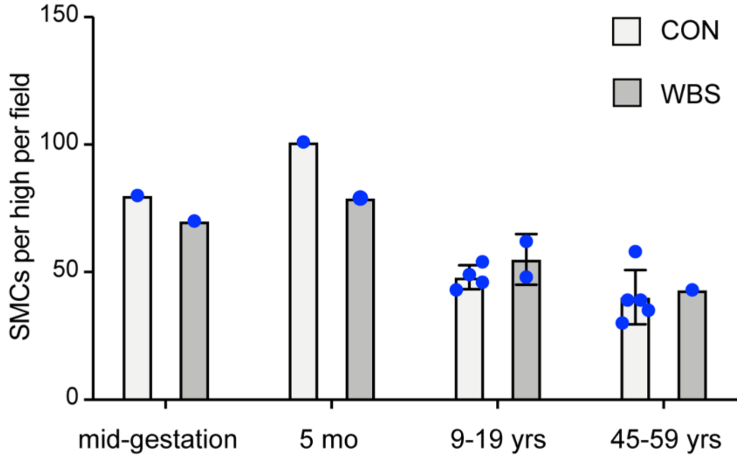

Supplemental Figure 16. Number of medial SMCs per high power field of aortic samples from WBS patients and controls stratified by age does not appear to differ substantially. Histograms represent number of medial SMCs per high power field from aortic sections of each WBS patient (n=5) and corresponding age-matched controls (n=11). The x-axis depicts age groups of donors as detailed in Supplemental Table 1. Data are averages ± SD.

Supplemental Table 1

WBS samples				Age-matched control samples			
**Sample**	**Sex**	**Age**	**Location**	**Sample**	**Sex**	**Age**	**Location**
WBS	N/A	mid-gestation	Ao	Control	N/A	mid-gestation	Ao
WBS	M	5 mo	Ao	Control	M	5 mo	Ao
WBS	F	9 y	Ao	Control	F	12 y	Ao
WBS	F	teen	Ao	Control	F	teen	Ao
				Control	M	19 y	Asc Ao
				Control	F	19 y	Asc Ao
WBS	M	46 y	Asc Ao	Control	M	45 y	Asc Ao
				Control	M	53 y	Asc Ao
				Control	M	59 y	Asc Ao
				Control	M	51 y	Asc Ao
				Control	F	53 y	Asc Ao

Age and sex information for human WBS patients and controls. This table provides the age and sex of WBS patients and controls that were utilized in Figure 8, J and K. The samples were categorized based on age-group. WBS samples were normalized to corresponding age-group matched controls. Asc Ao = Ascending aorta; Ao = aorta.

Supplemental Table 2

iPSC donor	Sex	Age
Control	M	newborn
SVAS	M	1.7 mo
WBS	M	10.5 mo

Age and sex information for human iPSC donors. This table provides the age and sex of iPSC donors utilized in Figures 1, G and H and 8, H and I.

Supplemental Table 3

Species	Gene	Forward primer	Reverse primer
Human	ELN	GTCGCAGGTGTCCCTAGTGT	GGTCCCCACTCCGTACTTG
Human	NOTCH1	CAATGTGGATGCCGCAGTTGTG	CAGCACCTTGGCGGTCTCGTA
Human	NOTCH2	ACAGTTGTGTCTGCTCACCAGGAT	GCGGAAACCATTCACACCGTTGAT
Human	NOTCH3	CCTAGACCTGGTGGACAAG	ACACAGTCGTAGCGGTTG
Human	NOTCH4	GCGGAGGCAGGGTCTCAACGGATG	AGGAGGCGGGATCGGAATGT
Human	HEY1	TTGGAGGCTTCCAGGTGGTA	GGCCCCGTTGGGAATG
Human	HEYL	CAGCCCTTCGCAGATGCAA	CCAATCGTCGCAATTCAGAAAG
Human	HES1	CCAGCCAGTGTCAACACGA	AATGCCGGGAGCTATCTTTCT
Human	JAG1	CGGGATTTGGTTAATGGTTATC	ATAGTCACTGGCACGGTTGTAGCAC
Human	JAG2	TACCAACGACTGCAACCCTC	GCACTCGTCGATGTTGATGC
Human	DLL1	CCTACTGCACAGAGCCGATCT	ACAGCCTGGATAGCGGATACAC
Human	DLL3	AATCGCCCTGAAGATGTAGACC	GCACCACCGAGCAAATACAA
Human	DLL4	ACTGTGCCCGTAACCCTTG	TGGAGAGGTCGGTGTAGCAG
Human	ITGB3	GGGGTAGGTTGGGAGAATGT	TCTGGGACAAAGGCTAAGGA
Human	DNMT1	ACCATGACAGGAAGAACGGC	CTTCCACGCAGGAGCAGA

Human	DNMT3A	GCTGGGAGTCCAGCCTCCGT	CCAGCCACTCGTCCCGCTTG
Human	DNMT3B	TAACAACGGCAAAGACCGAGGG	TCCTGCCACAAGACAAACAGCC
Human	ACTA2	CAAAGCCGGCCTTACAGAG	AGCCCAGCCAAGCACTG
Human	TAGLN	AAGCGCAGGAGCATAAGAGG	CTCTGTTGCTGCCCATCTGA
Human	CNN1	ATGTCCTCTGCTCACTTC	ATACTTCTGGGCCAGCTTGTT
Human	MYH11	CCTTGAGGAGAGGATTAGTGA	TTCCTTCTTTAGCCGCACTTC
Human	BIRC5	GCCAAGAACAAAATTGCAAAGG	TTTCTCCGCAGTTTCCTCAAA
Human	E2F1	CATCCCAGGAGGTCACTTCTG	GCCAAGAACAAAATTGCAAAGG
Human	18s rRNA	TAACGAACGAGACTCTGGCAT	CGGACATCTAAGGGCATCACAG
Mouse	Itgb3	GTGAGTGCGATGACTTCTCCTG	CAGGTGTCAGTGCGTGTAGTAC
Mouse	Notch3	GGTAGTCACTGTGAACACGAGG	CAACTGTCACCAGCATAGCCAG
Mouse	Elastin	TGCAGTACTGTAACCCCGTTC	CCAAAGAGCACACCAACAATC
Mouse	Gapdh	CATCACTGCCACCCAGAAGACTG	ATGCCAGTGAGCTTCCCGTTCAG
Mouse	18s rRNA	CCAAAGAGCACACCAACAATC	CCAAAGAGCACACCAACAATC

Primer pair sequences for qRT-PCR. This table shows primer pairs utilized for qRT-PCR analysis in the study with human and murine RNA samples.

SUPPLEMENTAL METHODS

Chromatin immunoprecipitation

haSMCs were transfected with siRNA targeted against ELN or NOTCH3 or Scr RNA and subjected to ChIP experiments as previously described (ref #70 in main text) with commercial reagents (SimpleChIP Enzymatic Chromatin IP Kit, Cell Signaling). Briefly, $1x10^6$ haSMCs were cross-linked and lysed. DNA was sheared to 500-1000 bp by sonication and incubated overnight at 4°C with antibodies against the intracellular domain of NOTCH3 (Santa Cruz, sc-515825) or isotype IgM (Cell Signaling) control. Protein G Dynabeads were used to pulldown the immunoprecipitated antibody-antigen complexes and incubated with Proteinase K to digest the protein. Recovered DNA was purified with spin columns and analyzed by qRT-PCR to assess enrichment at the *HES1*, *HEY1* and *ITGB3* gene promoters. The following forward and reverse primer pairs spanning canonical CSL binding motifs upstream of the transcription start site were used: *HES1* 5'- AAGGCCCCAAATCCAAACGA-3' and 5'-GCAGGTTTTTCGGAGCAAGG; *HEY1* 5'- AAAACAAGTGCTCCCCTTCC-3' and 5'-CATGCAGCCAGACTCGTTTC-3'; *ITGB3* 5'-GGCGAGAGAGGAGCAATAGT-3' and 5'-TCCGGCTTCTCTAGATCCCC-3'. The location of the PCR amplification product with *HES1* primers is chr3:194,135,445-194,135,526, with *HEY1* primers is chr8:80,680,967-80,681,063 and with *ITGB3* primers is chr17:45,331,003-45,331,085. All samples were performed in at least triplicate, from at least three independent experiments, and data were calculated by % input method.

Decellularization

haSMCs transfected with siRNA targeted against ELN or Scr RNA were cultured for 72 hours. Decellularization was performed by successive freeze-thaw cycles and detergent treatments. Briefly, transfected haSMCs were placed in -80°C for 30 minutes and then washed with PBS at room temperature for 30 minutes. This freeze-thaw cycle was repeated three times. Cells were then treated with 0.5% NP-40 for 30 minutes at room temperature and washed three times in PBS for 10 minutes. The resulting decellularized matrices were incubated at 37°C for 1 hour in EC media (ScienCell). Meanwhile, haECs were trypsinized and added to decellularized matrices at 90% confluency. After 24 hours, EC lysates were prepared in 1.5X Laemmli sample buffer and utilized for Western blotting.

Jag1 stimulation of haSMCs in culture

Human aortic SMCs between passages 3-6 were cultured on 12-well plates (Corning) coated with IgG, Fc fragment (R&D Systems) or recombinant human Jag1-FC chimera (rhJag1, R&D systems) as described previously (1). Briefly, culture plates were coated with Protein G (50 μg/mL, Zymed Laboratories) overnight followed by three washes in PBS, blocked with BSA (10 mg/mL in PBS) for 2 hours, washed three times in PBS and incubated with rhJag1 or IgG (5 μg/mL in 0.1% BSA) for 4 hours. Finally, the plates were washed in PBS and haSMCs were immediately seeded. After 24 hours, the cells were harvested for RNA isolation.

Lung EC isolation using anti-CD31-coated magnetic beads

Jag1$^{(flox/flox)}$ mice carrying no Cre or *Cdh5-Cre* were euthanized at P0.5, and lungs were perfused with PBS through the right heart ventricle. Harvested lungs were placed in

gentleMACS C Tubes (130-093-235, Miltenyi) containing 5 ml dispase (StemCell, 07923, 1 U/mL) and homogenized using the gentleMACS dissociator (Miltenyi, 130-093-235,) according to the manufacturer's guidelines. Homogenates were incubated at 37°C for 20 minutes, and the enzymatic reaction was stopped by adding 1 mL FBS with 1 mL DMEM (Gibco) and 2% (v/v) DNase (Thermo Fisher Scientific, 18047019). The lungs were further homogenized by gentleMACS dissociator and the resulting cell suspensions were sequentially filtered through a 100 µm and then 40 µm strainer before centrifugation at 4 °C at 1500 rpm for 10 minutes. The cell pellet was then resuspended in cold PBS to generate a single cell suspension. Lung ECs were isolated as previously described (ref #68 in main text). Briefly, sheep anti-rat-IgG Dynal magnetic beads (Invitrogen) were resuspended in PBS containing 0.1% FBS and incubated with mouse anti-rat CD31 monoclonal antibody (BD Biosciences, 1:250) overnight at 4°C, washed and stored 4°C. The lung single cell suspensions were incubated with these anti-CD31-coated beads for 20 minutes at room temperature and washed four times with sterile PBS. A magnet was used to separate cells bound to beads from unbound cells. Finally, the isolated lung ECs were lysed for RNA isolation and qRT-PCR as described below.

Detection of *Jag1* deletion efficiency

To determine *Jag1* deletion efficiency with *Cdh5-Cre* line, total RNA was isolated from lung ECs of *Jag1*$^{(flox,flox)}$ mice carrying no Cre or *Cdh5-Cre* at P0.5. For qRT-PCR analysis, the following forward and reverse primer pairs specific to the floxed region [exon 4, (2)] of *Jag1* were used: 5'- AAACACAGGGATTGCCCACT -3' and 5'- TGCAATCAGGACCCATCCAG -3'.

To elucidate *Jag1* deletion efficiency with *Acta2-CreER*^{T2}, pregnant dams bearing *Jag1*$^{(flox,flox)}$ with or without *Acta2-CreER*^{T2} were injected with 1 mg tamoxifen and concomitant 0.25 mg progesterone at E10.5 and newborn pups were collected. As described previously (3), aortas were dissected at P0.5 and incubated with 175 U/mL collagenase II (Thermofisher Scientific), 1.25 U/mL elastase (Worthington) in HBSS (Gibco) for 15 minutes at room temperature to facilitate stripping the adventitia. The adventitial layer was then carefully removed with tweezers under a microscope. The remaining medial layer was immediately collected in RNAlater (Sigma) and lysed in Trizol (Invitrogen) using stainless steel beads (Qiagen) and TissueLyser (Qiagen). RNA purification was performed using Direct-zol RNA microprep kit (Zymo Research) as per manufacturer's instructions followed by qRT-PCR analysis using *Jag1* exon 4 specific primers.

Sex determination

Y chromosome genotyping was utilized for determining the sex of neonatal pups at P0.5 as described previously (4). Briefly, tail biopsies were collected for genomic DNA isolation. The following primers were utilized to genotype for Y chromosome: mm-SRY-A 5'-TGGGACTGGTGACAATTGTC-3'and mm-SRY-B 5'- GAGTACAGGTGTGCAGCTCT-3' which generated a 402 bp length band using the following PCR conditions: 95°C for 4:30 minutes followed by 33 cycles of 95°C for 35 seconds, 50°C for 1 minute and 72°C for 1 minute. PCR products were terminated with a final extension at 72°C for 5 minute.

SUPPLEMENTAL REFERENCES

1. Jin S, Hansson EM, Tikka S, Lanner F, Sahlgren C, Farnebo F, et al. Notch signaling regulates platelet-derived growth factor receptor-beta expression in vascular smooth muscle cells. *Circ Res.* 2008;102(12):1483-91.
2. Kiernan AE, Xu J, and Gridley T. The Notch ligand JAG1 is required for sensory progenitor development in the mammalian inner ear. *PLoS Genet.* 2006;2(1):e4.
3. Ostriker AC, Xie Y, Chakraborty R, Sizer AJ, Bai Y, Ding M, et al. TET2 Protects Against Vascular Smooth Muscle Cell Apoptosis and Intimal Thickening in Transplant Vasculopathy. *Circulation.* 2021;144(6):455-70.
4. Lambert JF, Benoit BO, Colvin GA, Carlson J, Delville Y, and Quesenberry PJ. Quick sex determination of mouse fetuses. *J Neurosci Methods.* 2000;95(2):127-32.
